# The Role of lncRNA Polymorphisms in Digestive System Cancers: A Systematic Review and Meta-Analysis

**DOI:** 10.3390/cancers18121916

**Published:** 2026-06-12

**Authors:** Krisztina Varajti, Szimonetta Lohner, László Czina, Márk Kovács-Valasek, Afshin Zand, Tímea Varjas, István Kiss

**Affiliations:** 1 Department of Public Health Medicine, Medical School, University of Pécs, 7624 Pécs, Hungary; 2 Cochrane Hungary, University of Pécs Medical School, 7624 Pécs, Hungary; 3 PannonPharma Ltd., 7720 Pécsvárad, Hungary

**Keywords:** lncRNA, SNP, gastrointestinal cancer, HCC, GC, CRC, meta-analysis

## Abstract

Background: Gastrointestinal (GI) cancers, particularly colorectal, gastric, and liver cancers, account for a major global burden of incidence and mortality and remain important targets for genetic susceptibility research. Long non-coding RNAs (lncRNAs) can regulate gene expression and are increasingly studied in carcinogenesis. Numerous case–control studies have investigated associations between lncRNA polymorphisms and cancer risk, but findings are inconsistent. This study systematically evaluated the association between lncRNA single nucleotide polymorphisms (SNPs) and GI cancer susceptibility. Methods: A systematic literature search from Embase, Medline, Scopus, and Web of Science databases identified 174 potentially extractable studies. Eligible studies were case–control or cross-sectional studies published up to 8 May 2026; case reports, reviews, and meta-analyses were excluded. After screening for identical cancer type, identical SNP, and sufficient statistical data, only variants supported by at least three independent case–control studies were eligible for meta-analysis. Seven SNPs across six lncRNAs, comprising 23 studies (15,131 cases and 20,969 controls), were selected. Because of the limited number of eligible studies, subgroup analyses could not be performed consistently. Odds ratios (ORs) with 95% confidence intervals (CIs) were assessed under allelic, dominant, and recessive genetic models using fixed- or random-effects models according to heterogeneity. Results: In the primary analyses restricted to homogenous Chinese populations, H19 rs3024270 was significantly associated with hepatocellular carcinoma under allelic (OR = 1.22, 95% CI: 1.05–1.42, *p* = 0.01) and dominant models (OR = 1.22, 95% CI: 1.03–1.45, *p* = 0.02). Exploratory analyses including mixed populations identified additional associations, with the strongest observed for MEG3 rs7158663 and colorectal cancer, showing significant risk elevation under allelic (OR = 1.42, 95% CI: 1.25–1.63, *p* < 0.00001), dominant (OR = 1.42, 95% CI: 1.20–1.68, *p* < 0.0001), and recessive models (OR = 1.98, 95% CI: 1.46–2.68, *p* < 0.0001). PRNCR1 rs16901946 showed a significant association with gastric cancer under the dominant model (OR = 1.20, 95% CI: 1.02–1.41, *p* = 0.03), while GAS5 rs145204276 demonstrated a recessive-model association with gastric cancer (OR = 1.30, 95% CI: 1.16–1.46, *p* < 0.0001). In contrast, GAS5 rs145204276 in colorectal cancer; H19 rs2839698 and MALAT1 rs619586 in hepatocellular carcinoma yielded heterogeneous or unstable pooled estimates. Findings should be interpreted cautiously due to the limited number of studies, heterogeneity, and potential publication bias. Conclusions: Among the primary analyses, H19 rs3024270 showed the most consistent association with HCC susceptibility. Exploratory analyses identified candidate variants, including MEG3 rs7158663, PRNCR1 rs16901946, and GAS5 rs145204276. Population-specific effects and study heterogeneity remain important limitations. PROSPERO registration number for this study: CRD42023389742.

## Simple Summary

Gastrointestinal cancers, including colorectal, stomach, and liver cancers, are among the leading causes of cancer-related mortality worldwide. Identifying inherited genetic factors that influence cancer risk may improve prevention, early detection, and future treatment strategies. In recent years, increasing attention has focused on long non-coding RNAs, molecules that help regulate gene activity and may contribute to cancer development. Many studies have examined whether small genetic differences in these molecules are linked to cancer risk, but their results have often been conflicting. In this study, we combined data from multiple previous investigations to provide a clearer overall picture. Our findings suggest that several genetic variants in long non-coding RNAs are associated with a higher risk of gastrointestinal cancers, particularly colorectal, liver, and stomach cancer. These results may help guide future genetic research and support the development of more personalized cancer risk assessment.

## 1. Introduction

Cancers of the gastrointestinal (GI) tract, mainly colorectal, gastric, and liver cancers represent a major contribution to the global cancer burden, both in terms of incidence and mortality. According to the WHO GLOBOCAN 2022 estimates, colorectal cancer (CRC) is the third most frequently diagnosed cancer worldwide, accounting for 9.6% of all new cancer cases, and ranks second in cancer-related mortality with 9.3% of all deaths [1]. While gastric and liver cancers show lower incidence rates (4.8% and 4.3%, respectively), they are responsible for an overly higher ratio of cancer-related deaths, contributing to 6.8% and 7.8% of the total mortality, respectively [1]. Dietary and lifestyle changes, particularly the increasing consumption of energy-dense, high-fat diets, have also been implicated in GI carcinogenesis through their effects on the gut microbiota. The bidirectional interaction between the intestinal microbiome and host gene regulation is increasingly recognized as an important factor in GI cancer development [2,3]. These observations underscore the urgent need for a better understanding of the genetic and molecular factors underlying GI cancers to improve early detection and prevention strategies.

Long non-coding RNAs (lncRNAs) are RNA molecules at least 200 nucleotides long [4]. They can be classified into several groups based on their genomic localization, and their expression exhibits a high level of tissue specificity [5,6,7]. Over the years, more than 35.000 human lncRNA genes and more than 190.000 lncRNA transcripts have been annotated in the human genome [7,8,9,10]. LncRNAs are involved in regulation at all levels of transcription. They are, with overlapping functionality, signaling molecules of specific cellular states, including cell growth and morphological phases [11,12,13], and thus can not only help to identify pathological changes in cells resulting from oncogenesis, but can also provide crucial information for prognosis and therapeutic decisions as biomarkers [14,15,16,17,18,19]. Accumulating evidence indicates that lncRNAs play important roles in the development and progression of GI cancer. Dysregulated expression of several lncRNAs has been implicated in key cancer-related processes, including cell proliferation, apoptosis, invasion, metastasis and chemoresistance in colorectal, gastric and hepatocellular carcinomas [17]. Certain lncRNAs such as H19, HOTAIR, MALAT1 and CCAT have been proposed as potential diagnostic and prognostic biomarkers in GI cancers due to their involvement in tumor growth and disease progression [20,21,22]. Recent studies have further highlighted that non-coding RNA networks extend beyond lncRNAs and include other regulatory RNA types, such as circular RNAs (circRNAs), which participate in tumor initiation, progression, and therapeutic response across multiple cancer types [23]. In addition, advances in molecular oncology continue to emphasize the growing importance of RNA-based regulatory mechanisms and biomarkers for improving cancer diagnosis, prognosis, and personalized treatment strategies [24]. Several meta-analyses have emphasized associations between single nucleotide polymorphisms (SNPs) in lncRNAs and susceptibility to various cancer types [25], including but not limited to gastric [26,27], gastrointestinal [28,29], breast [30,31] and lung [32,33] cancers. These findings highlight the possible role of lncRNA-related genetic variants in cancer pathogenesis and progression. However, the available evidence remains incomplete and inconsistent, as many reported associations are based on limited numbers of studies, small sample sizes, heterogeneous populations, or conflicting results across individual investigations. Therefore, we conducted the present systematic review and meta-analysis to offer a more comprehensive and updated evaluation of recurrently studied lncRNA polymorphisms in gastrointestinal cancers. Our aim was to synthesize and critically evaluate the available literature to refine the potential impact of lncRNA SNPs to GI cancer risk. By aggregating data across multiple independent studies, we wanted to provide a more robust estimation of the effect sizes and to identify consistent genetic markers that may serve as feasible biomarkers for GI cancer susceptibility.

## 2. Materials and Methods

### 2.1. Search Strategy and Data Extraction

A comprehensive literature search was performed on Embase, Medline (via Ovid), Scopus, and Web of Science databases using combinations of the keywords gastrointestinal, cancer, lncRNA, and SNP. Detailed search strategy is defined in Supplementary Figure S1. The systematic review and meta-analysis was registered in PROSPERO (ID: CRD42023389742) and was conducted in accordance with the Preferred Reporting Items for Systematic Reviews and Meta-Analyses (PRISMA) 2020 guidelines [34]. PRISMA 2020 checklist and checklist for abstracts are provided in Supplementary Tables S1 and S2. The full protocol can be accessed at https://www.crd.york.ac.uk/PROSPERO/view/CRD42023389742 (accessed on 4 June 2026). The inclusion criteria were case–control and cross-sectional studies published up to 8 May 2026. Case reports, review articles, and meta-analyses were excluded. All screening and data extraction steps were conducted independently by two authors. Covidence platform was used for the screening process (Covidence systematic review software, Veritas Health Innovation, Melbourne, Australia. Available at www.covidence.org, accessed on 4 June 2026). The reference list of the included studies were also screened manually by two independent authors to identify additional potentially eligible studies for inclusion. The following information was collected from each study by two independent authors, manually: first author, year of publication, cancer type, lncRNA, SNP, ethnicity, number of cases and controls, total sample size, genotype distribution, genotyping method and sample type.

### 2.2. Statistical Analysis

If at least three studies were available for the same lncRNA SNP in a specific gastrointestinal cancer type, a meta-analysis was conducted to estimate the pooled genetic effect. For each lncRNA SNP, three genetic models—dominant, recessive, and allelic—were evaluated. Given the marked inconsistency across studies in some cases, exploratory sensitivity analyses were performed to assess the influence of individual datasets. Experimental meta-analyses were performed for mixed population pools. Pooled effect sizes were expressed as odds ratios (ORs) with corresponding 95% confidence intervals (CIs), calculated using the Mantel-Haenszel method. A random-effects model was applied in cases of substantial heterogeneity; otherwise; the analysis was performed using a fixed-effects model. Potential publication bias was assessed by visual examination of Funnel plots for meta-analyses including at least three studies. Given the limited number of eligible studies, the evaluation of subgroup analyses was not consistently feasible. All statistical analyses were performed using Review Manager (RevMan) version 5.4.1 (Cochrane, London, UK). Hardy–Weinberg equilibrium (HWE) in control groups was re-assessed using the χ^2^ test with IBM SPSS Statistics version 27 (IBM Corporation, Armonk, NY, USA). The quality of the included studies was evaluated using the Newcastle-Ottawa Scale (NOS).

## 3. Results

### 3.1. Search Results, Characteristics of the Included Studies and Publication Bias Assessments

**Figure 1:**
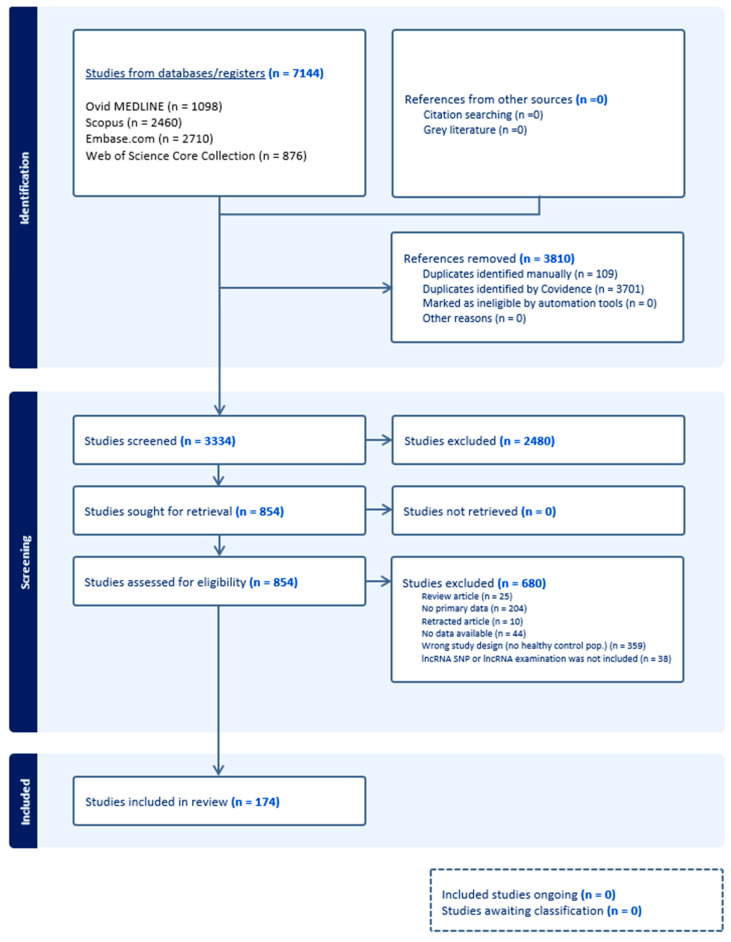
Flow chart of literature search and study selection according to PRISMA 2020, derived from Covidence.

Following a systematic literature review conducted in accordance with our predefined search strategy, 174 studies were initially evaluated as potentially data-extractable for quantitative synthesis (Figure 1). We then performed an initial screening based on cancer type and investigated SNP to identify studies with sufficiently comparable datasets suitable for statistical pooling. For the majority of identified variants, each SNP had been investigated in only a single study, which precluded formal meta-analysis. To ensure that pooled estimates were statistically meaningful, sufficiently powered, and methodologically reliable, meta-analyses were restricted to SNPs supported by at least three independent case–control studies investigating the same cancer type, thereby maximizing clinical and biological comparability across pooled datasets (Table 1). Genotyping methods included targeted analyses as qPCR (SYBR), TaqMan RT-qPCR, RT-PCR, PCR-RFLP, Sequenom MassARRAY, KASP, microarray and direct sequencing. Sample types were predominantly peripherial or venous blood specimens (*n* = 21), while a smaller number of studies used tissue samples (*n* = 3), serum samples (*n* = 2), or both blood and tumor tissue samples (*n* = 1). The sample type was not available in three studies. The studies performed by Motawi et al. and Wei et al. (as well as H19 rs217727) were excluded from meta-analyses because the genotype distribution in the control groups significantly deviated from Hardy–Weinberg equilibrium (*p* = 0.013 and *p* < 0.001, respectively). The NOS scores ranged from 7 to 8, indicating moderate-to-high overall study quality (Supplementary Table S3). Most studies achieved high scores for case definition, control selection, and genotyping methodology. No study was classified as low quality. Following this screening process, only 7 SNPs across 6 distinct lncRNAs fulfilled the criteria for meta-analysis, corresponding to 23 case–control studies included in quantitative synthesis (Table 1). However, considerable between-study differences were observed in the ethnic composition of investigated populations, which may influence allele frequencies and genetic effect estimates, thereby limiting direct comparability. Therefore, primary meta-analyses in Chinese populations were performed only for SNPs within the H19, MALAT1 and HOTAIR lncRNAs. In contrast, exploratory meta-analyses involving partially mixed populations were conducted for SNPs within GAS5, PRNCR1 and MEG3. In these analyses, two of the three available studies were derived from Chinese populations, while one study represented a different ethnic background. Accordingly, these findings should be interpreted with appropriate caution due to population heterogeneity. Visual inspection of the Funnel plots (Supplementary Figure S2) did not reveal substantial asymmetry for any of the analyzed polymorphisms. However, because all comparisons included only three studies, the ability of Funnel plots to detect publication bias was limited, and the findings should therefore be interpreted with caution.

### 3.2. Primary Evidence from Chinese Population

#### 3.2.1. H19 rs2839698 and Hepatocellular Carcinoma (HCC) Risk in Chinese Population

**Figure 2:**
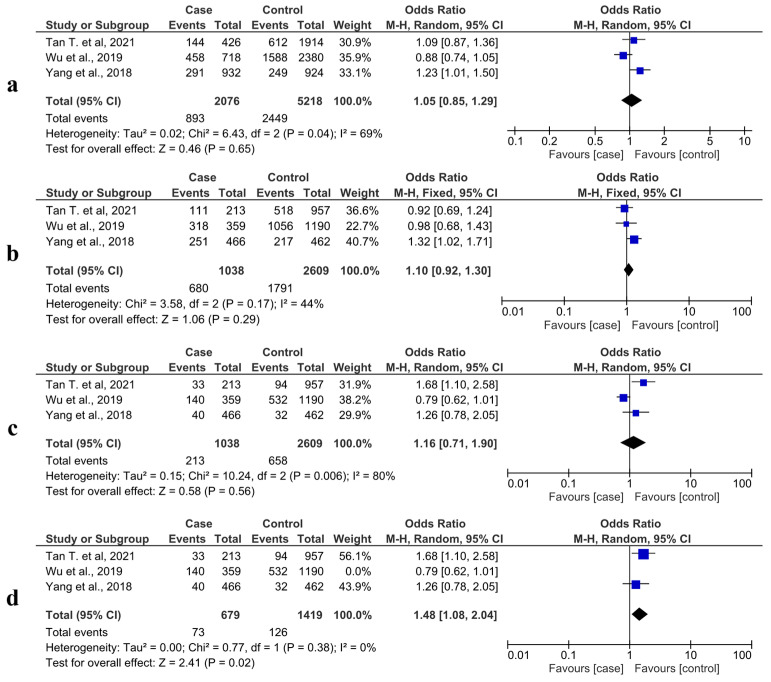
Forest plots of the association between H19 rs2839698 polymorphism and HCC risk under different genetic models: (**a**) allelic model; (**b**) dominant model; (**c**) recessive model; and (**d**) recessive model after sensitivity analysis excluding the study by Wu et al. [48]. The studies included for these analyses: Tan T. et al., Wu et al. and Yang et al. [47,48,49].

Pooled analysis under the allelic model using a random-effects approach demonstrated no statistically significant association between the risk allele and HCC risk (OR = 1.05, 95% CI: 0.85–1.29, *p* = 0.65), with considerable between-study heterogeneity (I^2^ = 69%) (Figure 2a). Similarly, under the dominant model, no significant association was observed (OR = 1.10, 95% CI: 0.92–1.30, *p* = 0.29), with moderate heterogeneity (I^2^ = 44%) (Figure 2b). Under the recessive model, the primary random-effects meta-analysis also showed no significant association (OR = 1.16, 95% CI: 0.71–1.90, *p* = 0.56), although high heterogeneity was present (I^2^ = 80%) (Figure 2c). Sequential exclusion analyses identified the Wu et al. [48] study as the principal source of heterogeneity. After removal of this dataset, heterogeneity was eliminated under the recessive model (I^2^ = 0%), and a statistically significant association emerged between rs2839698 and increased HCC susceptibility (OR = 1.48, 95% CI: 1.08–2.04, *p* = 0.02) (Figure 2d). This finding suggests that individuals homozygous for the variant genotype may have a higher risk of developing HCC. However, this result should be interpreted cautiously, as exclusion of Wu et al. reduced the analysis to only two remaining studies, thereby limiting statistical power and the robustness of the pooled estimate. A similar trend was observed under the allelic model after exclusion of Wu et al., although statistical significance was only borderline.

#### 3.2.2. H19 rs3024270 and HCC Risk in Chinese Population

**Figure 3:**
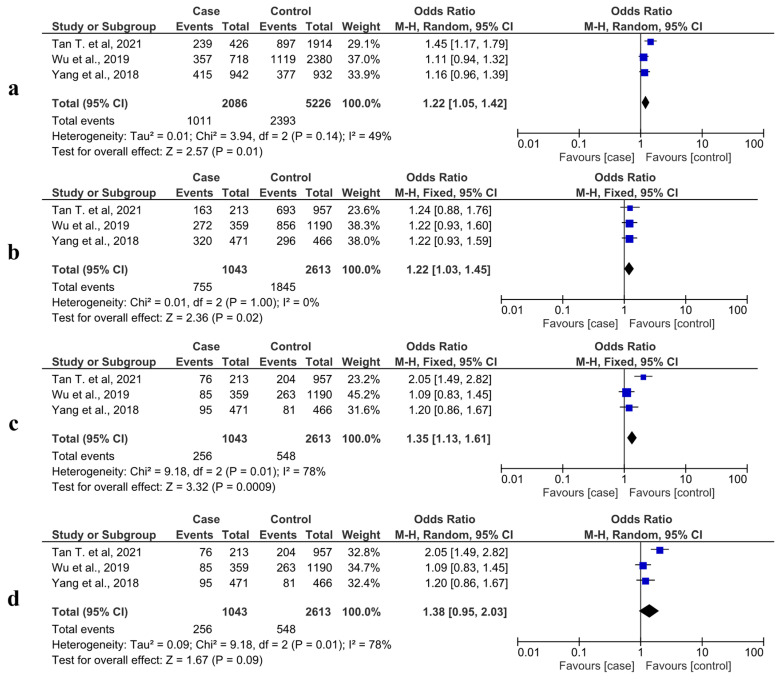
Forest plots of the association between H19 rs3024270 polymorphism and HCC risk under different genetic models: (**a**) allelic model; (**b**) dominant model; (**c**) recessive model using a fixed-effect model; and (**d**) recessive model using a random-effects model [47,48,49].

Across all included studies, the direction of effect was consistent, with study-specific estimates indicating increased risk among carriers of the variant allele. Under the allelic model, pooled analysis using a random-effects approach demonstrated a statistically significant association between rs3024270 and increased HCC susceptibility (OR = 1.22, 95% CI: 1.05–1.42, *p* = 0.01), with moderate between-study heterogeneity (I^2^ = 49%) (Figure 3a). Under the dominant model, a significant association was also observed (OR = 1.22, 95% CI: 1.03–1.45, *p* = 0.02), with no evidence of heterogeneity across studies (I^2^ = 0%) (Figure 3b). This indicates that carriers of at least one variant allele had a higher risk of HCC compared with individuals with the wild-type genotype. Under the recessive model, the fixed-effect analysis suggested a significant association (OR = 1.35, 95% CI: 1.13–1.61, *p* = 0.0009); however, extensive heterogeneity was present (I^2^ = 78%) (Figure 3c). Accordingly, the more appropriate random-effects model was applied, which attenuated the association and rendered it no longer statistically significant, although a positive trend remained (OR = 1.38, 95% CI: 0.95–2.03, *p* = 0.09) (Figure 3d).

#### 3.2.3. HOTAIR rs4759314 and Gastric Carcinoma (GC) Risk in Chinese Population

**Figure 4:**
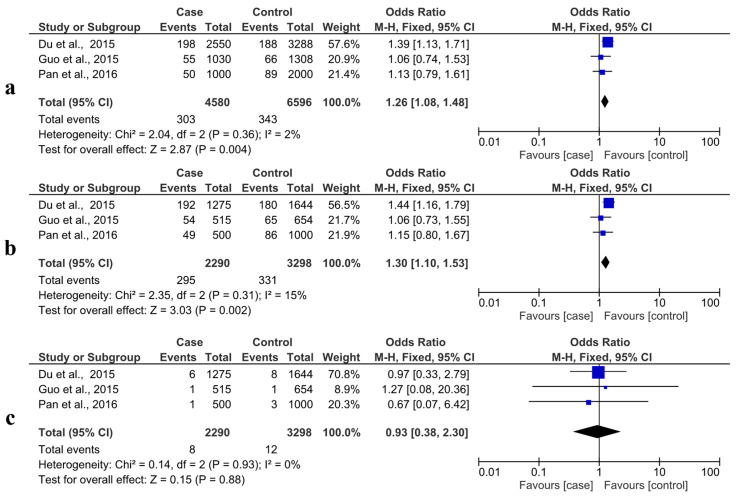
Forest plots of the association between HOTAIR rs4759314 polymorphism and GC risk under different genetic models: (**a**) allelic model; (**b**) dominant model; and (**c**) recessive model [50,52,53].

Under the allelic model, pooled fixed-effect analysis demonstrated a significant association between rs4759314 and increased GC susceptibility (OR = 1.26, 95% CI: 1.08–1.48, *p* = 0.004), with negligible between-study heterogeneity (I^2^ = 2%) (Figure 4a). Under the dominant model, a similarly significant association was observed (OR = 1.30, 95% CI: 1.10–1.53, *p* = 0.002), with low heterogeneity across studies (I^2^ = 15%) (Figure 4b). This indicates that individuals carrying at least one variant allele had a significantly higher risk of GC compared with wild-type homozygotes. In contrast, no significant association was detected under the recessive model (OR = 0.93, 95% CI: 0.38–2.30, *p* = 0.88), and no heterogeneity was observed (I^2^ = 0%) (Figure 4c). However, the number of homozygous variant carriers was very low across studies, resulting in low statistical power and broad confidence intervals.

#### 3.2.4. MALAT1 rs619586 and HCC Risk in Chinese Population

**Figure 5:**
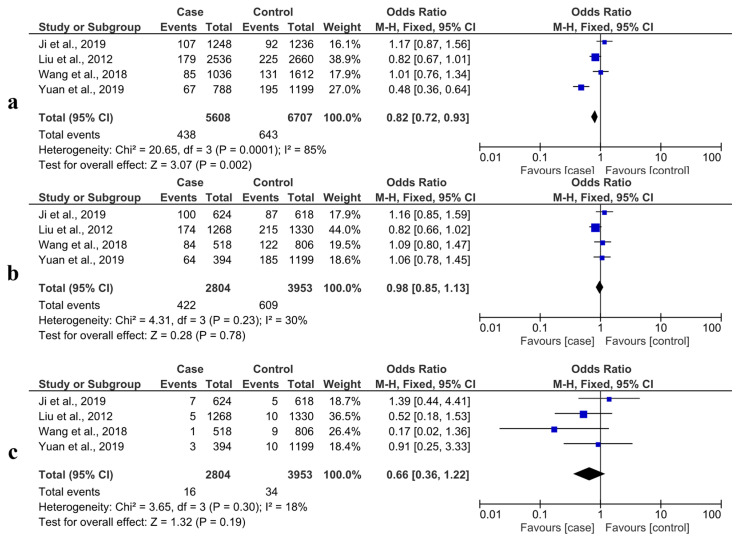
Forest plots of the association between MALAT1 rs619586 polymorphism and hepatocellular carcinoma risk under different genetic models: (**a**) allelic; (**b**) dominant; (**c**) recessive [55,56,57,58].

Under the allelic model, fixed-effect analysis demonstrated a significant association between rs619586 and HCC susceptibility (OR = 0.82, 95% CI: 0.72–0.93, *p* = 0.002), although substantial between-study heterogeneity was observed (I^2^ = 85%) (Figure 5a). Under the dominant model, fixed-effect analysis demonstrated no significant association between rs619586 and HCC susceptibility (OR = 0.98, 95% CI: 0.85–1.13, *p* = 0.78), with low-to-moderate heterogeneity across studies (I^2^ = 30%) (Figure 5b). Similarly, under the recessive model, no significant association was observed (OR = 0.66, 95% CI: 0.36–1.22, *p* = 0.19), with low heterogeneity (I^2^ = 18%) (Figure 5c). However, the number of homozygous variant carriers was very low across studies, resulting in limited statistical power and broad confidence intervals.

### 3.3. Exploratory Evidence from Partially Mixed Population

#### 3.3.1. GAS5 rs145204276 and Colorectal Cancer (CRC) Risk in Mixed Populations

Three case–control studies were eligible for exploratory meta-analysis, including two Chinese cohorts and one Romanian cohort [36,37,38]. Across all inheritance models, substantial to extreme between-study heterogeneity was observed (allelic: I^2^ = 98%; dominant: I^2^ = 94%; recessive: I^2^ = 90%), indicating marked inconsistency among studies. Under the allelic model, the two Chinese studies showed protective associations (Zheng et al., OR = 0.79; Zhu et al., OR = 0.32), whereas the Romanian study (Mirea et al.) demonstrated an opposite, risk-increasing effect (OR = 1.99). Although the pooled estimate suggested an overall protective association (OR = 0.63, 95% CI: 0.57–0.69, *p* < 0.00001), this result was driven by highly conflicting individual effects and should be interpreted cautiously. Under the dominant model, study-specific findings remained inconsistent as Zheng et al. showed a protective effect (OR = 0.75), while Zhu et al. (OR = 1.44) and Mirea et al. (OR = 2.13) indicated increased risk. The pooled estimate was null (OR = 1.00, 95% CI: 0.89–1.12, *p* = 0.98). Similarly, under the recessive model, Zheng et al. suggested a protective association (OR = 0.72), whereas Zhu et al. (OR = 1.81) and Mirea et al. (OR = 2.52) showed risk-increasing tendencies. No significant pooled association was detected (OR = 1.05, 95% CI: 0.87–1.28, *p* = 0.61). All corresponding figures are provided in Supplementary Figure S3.

#### 3.3.2. GAS5 rs145204276 and GC Risk in Mixed Populations

**Figure 6:**
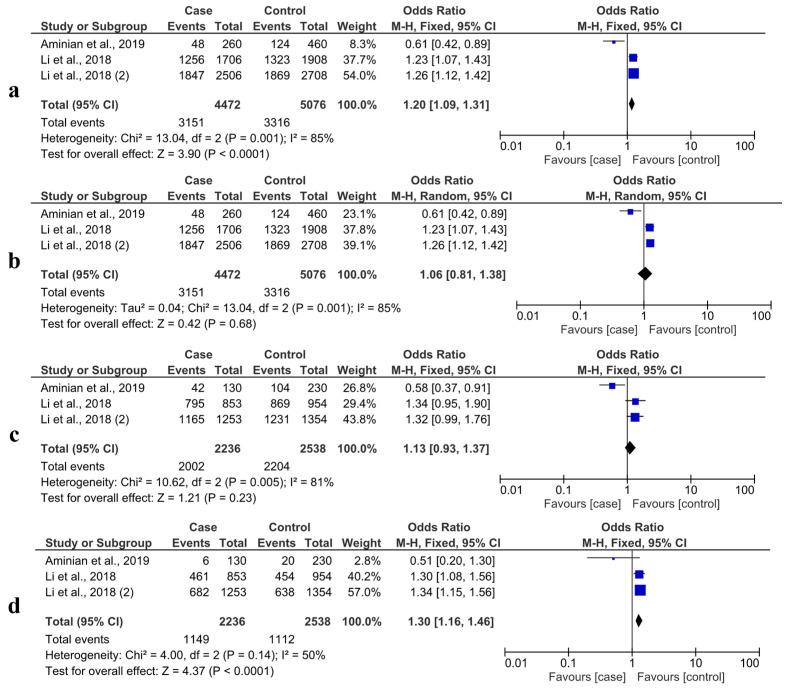
Forest plots of the association between GAS5 rs145204276 polymorphism and gastric cancer risk under different genetic models: (**a**) allelic model using a fixed-effect model; (**b**) allelic model using a random-effects model; (**c**) dominant model; and (**d**) recessive model [39,40,41].

The exploratory pooled analysis included three case–control studies: two from Chinese and one from Iranian cohort. Across models, study-specific estimates showed a consistent risk-increasing effect in the two Chinese cohorts, whereas the smaller Iranian cohort demonstrated an opposite, protective direction of effect. Under the allelic model, the fixed-effect analysis suggested a significant association (OR = 1.20, 95% CI: 1.09–1.31, *p* < 0.0001); however, high heterogeneity was present (I^2^ = 85%) (Figure 6a). After application of the random-effects model, the association was no longer significant (OR = 1.06, 95% CI: 0.81–1.38, *p* = 0.68) (Figure 6b), indicating that the fixed-effect result was not robust. Similarly, under the dominant model, no significant overall association was observed (OR = 1.13, 95% CI: 0.93–1.37, *p* = 0.23), with considerable heterogeneity (I^2^ = 81%) (Figure 6c). In contrast, under the recessive model, a significant association was identified between rs145204276 and increased GC susceptibility (OR = 1.30, 95% CI: 1.16–1.46, *p* < 0.0001), with moderate heterogeneity (I^2^ = 50%) (Figure 6d). This positive effect was primarily driven by the two Chinese cohorts, while the Iranian cohort contributed minimal statistical weight and showed an opposite-direction estimate.

#### 3.3.3. PRNCR1 rs16901946 and GC Risk in Mixed Populations

**Figure 7:**
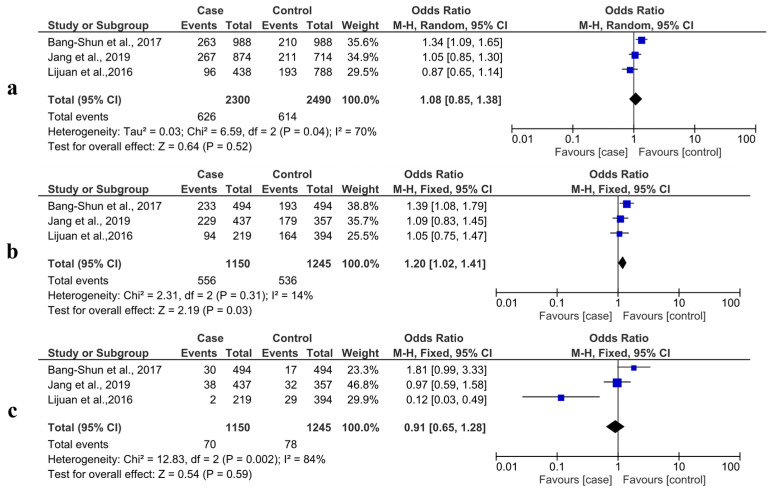
Forest plots of the association between PRNCR1 rs16901946 and GC risk under different genetic models: (**a**) allelic model; (**b**) dominant model; and (**c**) recessive model [42,43,44].

Three studies met the inclusion criteria for exploratory meta-analysis, consisting of two Chinese cohorts and one Korean cohort. Under the allelic model, no significant association was observed using a random-effects approach (OR = 1.08, 95% CI: 0.85–1.38, *p* = 0.52), with broad between-study heterogeneity (I^2^ = 70%) (Figure 7a). Study-specific estimates were inconsistent, including one protective Chinese cohort, one neutral Korean cohort, and one risk-increasing Chinese cohort. In contrast, under the dominant model, a significant association was identified between rs16901946 and increased GC susceptibility (OR = 1.20, 95% CI: 1.02–1.41, *p* = 0.03), with low heterogeneity (I^2^ = 14%) (Figure 7b). All three studies showed effect estimates in the same direction (OR > 1), indicating that carriers of at least one variant allele had a higher risk of GC compared with wild-type homozygotes. Under the recessive model, no significant association was detected (OR = 0.91, 95% CI: 0.65–1.28, *p* = 0.59), with marked heterogeneity across studies (I^2^ = 84%), reflecting highly conflicting study-specific results (Figure 7c).

#### 3.3.4. MEG3 rs7158663 and CRC Risk in Mixed Populations

**Figure 8:**
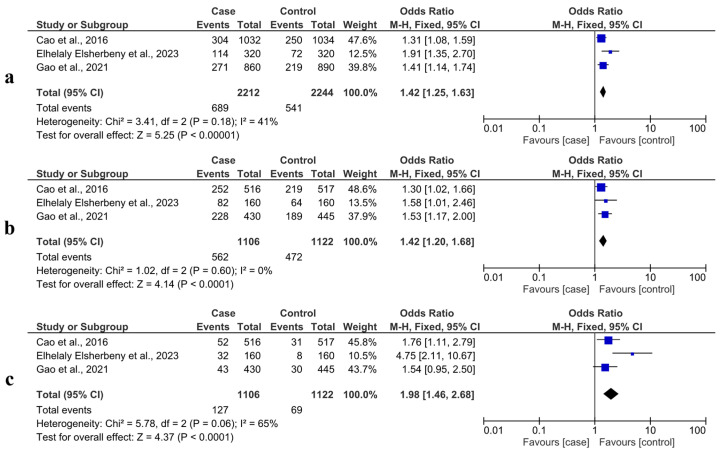
Forest plots of the association between MEG3 rs7158663 polymorphism and colorectal cancer risk under different genetic models: (**a**) allelic model; (**b**) dominant model; and (**c**) recessive model [59,60,61].

Three eligible case–control studies were identified, representing two Chinese cohorts and one Egyptian cohort. Because of the mixed ethnic composition, pooled analyses were interpreted with appropriate caution; however, study-specific effect estimates were directionally consistent across all included populations. Under the allelic model, rs7158663 was significantly associated with increased CRC risk (OR = 1.42, 95% CI: 1.25–1.63, *p* < 0.00001), with low-to-moderate heterogeneity (I^2^ = 41%) (Figure 8a). All three studies demonstrated risk-increasing effects. Similarly, under the dominant model, carriers of at least one variant allele had a significantly elevated CRC risk (OR = 1.42, 95% CI: 1.20–1.68, *p* < 0.0001), with no detectable heterogeneity (I^2^ = 0%) (Figure 8b). Study-specific estimates were highly concordant across the Chinese and Egyptian cohorts. The strongest association was observed under the recessive model, where homozygous variant carriers showed an almost twofold increased CRC risk (OR = 1.98, 95% CI: 1.46–2.68, *p* < 0.0001), with mild heterogeneity (I^2^ = 65%) (Figure 8c). All included studies showed effect estimates in the same risk-increasing direction.

## 4. Discussion

This meta-analysis identified several lncRNA polymorphisms associated with GI cancer susceptibility, although the strength and consistency of evidence varied clearly across cancer types. Notably, most of the included studies used peripherial or venous blood samples for genotyping, representing a liquid biopsy-based approach, while only a minority relied on tissue-derived specimens. Overall, the most robust signals were observed for MEG3 rs7158663 in CRC, H19 rs3024270 in HCC, HOTAIR rs4759314 in GC, and PRNCR1 rs16901946 in GC. In contrast, H19 rs2839698, MALAT1 rs619586 and GAS5 rs145204276 showed heterogeneous or population-dependent effects, highlighting the complexity of lncRNA-based cancer genetics.

In hepatocellular carcinoma, all meta-analyses were based on studies conducted in Chinese populations. For H19 rs2839698, no significant overall association with HCC was observed. Although sensitivity analysis yielded a significant association under the recessive model after exclusion of one heterogenous dataset, this result was based on only two remaining studies and therefore cannot be considered as robust evidence of an association. Consequently, the finding should be regarded as exploratory and interpreted with caution until further validation. Several previous meta-analyses have also examined the relationship between H19 rs2839698 and cancer susceptibility. Early analyses by Chu et al. [62] and Li et al. [63] reported inconsistent overall results, although subgroup analyses suggested increased risk particularly for digestive system cancers. Similarly, Hashemi et al. observed stronger associations in gastrointestinal malignancies [64]. More recent and larger meta-analyses by Liu et al. [65], Yuan et al. [66], and Yang et al. [67] consistently demonstrated that rs2839698 is associated with elevated overall cancer susceptibility, particularly in Asian populations and under recessive or homozygous genetic models. Several of these analyses also identified stronger effects in digestive system malignancies, including hepatocellular carcinoma, supporting the possibility that rs2839698 may contribute to HCC susceptibility in a population- and model-dependent manner.

By contrast, H19 rs3024270 showed a more consistent association with HCC, particularly under allelic and dominant models, indicating that a single risk allele may be sufficient to influence susceptibility. As all included studies were performed in Chinese Han populations, internal consistency was good, although external generalizability remains uncertain. Previous meta-analyses evaluating H19 polymorphisms in cancer susceptibility have also included rs3024270, although the available evidence remains more limited than for rs2839698. While some analyses reported no significant overall association for rs3024270 [65], subgroup analyses suggested possible effects in specific populations or genetic models [68]. However, Yang et al. [67] and Wang et al. [69] demonstrated a significant association between rs3024270 and overall, population-based cancer susceptibility, supporting a potential contribution of this variant to carcinogenesis. However, tumour-specific evidence for HCC remains relatively insufficient, and further studies in larger and ethnically diverse cohorts are warranted.

Similarly, MALAT1 rs619586 demonstrated no significant association with HCC susceptibility under dominant or recessive models. Study-specific effect estimates were inconsistent across the four included studies, with some studies showing a protective effect and the remaining studies demonstrating neutral or mildly risk-increasing associations. Substantial heterogeneity was observed under the allelic model, largely driven by the protective-effect study. The dominant and recessive models likewise demonstrated no evidence of association, suggesting that rs619586 is unlikely to play a key role in HCC susceptibility according to the currently available literature. These findings are in line with the meta-analysis by Ni et al., which reported that although rs619586 was associated with overall cancer susceptibility, no significant association was observed specifically for hepatocellular carcinoma in subgroup analyses [70]. This may indicate that the effect of rs619586 is cancer-type dependent and less relevant in HCC than in other malignancies.

In gastric cancer, HOTAIR rs4759314 demonstrated a stable positive association, strongest under the dominant model and with minimal heterogeneity. Similar findings were reported by Qi et al., whose subgroup analysis identified a significant association between rs4759314 and gastric cancer susceptibility under allelic, dominant, and heterozygous models, with little heterogeneity within the gastric cancer subgroup [71]. Together these findings collectively support a potential role of HOTAIR rs4759314 in gastric carcinogenesis.

PRNCR1 rs16901946 similarly showed a modest but consistent dominant-model association, supported by low heterogeneity across East Asian cohorts. Comparable findings have been reported in a previous meta-analysis investigating PRNCR1 polymorphisms and cancer susceptibility, in which Du et al. confirmed the contribution of rs16901946 to cancer susceptibility across multiple genetic models [72].

In contrast, GAS5 rs145204276 was associated with gastric cancer primarily under the recessive model, with the signal largely driven by Chinese cohorts, while the Iranian study showed an opposite effect direction. This suggests possible ancestry-specific effects. Previously Cai et al. confirmed a significant correlation between rs145204276 and gastric cancer susceptibility [73], whereas Gao et al. found no significant association with overall cancer risk, although stratified analyses suggested that rs145204276 may act as a protective factor in gastric cancer [74]. Both investigated Asian populations. Together, these findings indicate that the effect of GAS5 rs145204276 may differ according to ethnic background and tumour type.

For colorectal cancer, MEG3 rs7158663 was the strongest overall finding, with significant associations across all inheritance models and the largest effect under the recessive model. Concordant risk estimates across Chinese and Egyptian cohorts support a potential biological effect, although mixed-population pooling warrants cautious interpretation. Similar findings have been described in previous meta-analyses. Gao et al. demonstrated significant associations between rs7158663 and colorectal as well as gastric cancer risk [59], while Wang et al. also observed increased susceptibility particularly for colorectal cancer, but not liver cancer [75]. More recently, Hu et al. confirmed that the rs7158663 A allele was associated with elevated overall cancer risk, especially in East Asian and Middle Eastern populations [76]. These findings support a possible role of MEG3 rs7158663 in colorectal carcinogenesis while also suggesting possible population-specific effects.

Conversely, GAS5 rs145204276 yielded conflicting results across studies and models, possibly reflecting population stratification, methodological differences, or inconsistent in/del allele coding. Gao et al. found no significant relationship between rs145204276 and overall cancer susceptibility, although subgroup analyses suggested a protective effect in gastric cancer [74], whereas Cai et al. reported significant associations in gastrointestinal malignancies [73]. These discrepancies may partly result from ethnic differences or heterogeneous study designs.

Because GAS5 rs145204276 is an insertion/deletion polymorphism, differences in allele designation and genotype nomenclature across studies may potentially influence the interpretation of pooled results. To address this issue, we reviewed the genotype coding reported in all included studies of this SNP. No obvious inconsistencies in a genotype coding were identified. Notably, conflicting associations were observed even among Chinese studies, indicating that the observed heterogeneity is unlikely to be explained solely by allele-coding discrepancies, it is more likely influenced by population-specific genetic effects.

Nevertheless, several important limitations must be emphasized. First, the number of available studies was small, reducing the statistical power and overall robustness of the pooled estimates. The limited sample size also restricted the possibility of conducting more detailed subgroup analyses and may have reduced the ability to detect modest genetic effects. Second, potential population-specific effects and residual heterogeneity across analyses may have influenced the observed associations. Most of the included studies were conducted in Chinese populations, resulting in an ethnic imbalance that may limit the generalizability of the findings to other populations. Differences in allele frequencies, linkage disequilibrium patterns, environmental exposures, and genetic background across ethnic groups may contribute to variation in SNP-disease associations. Furthermore, population stratification may act as a potential confounder in genetic association studies and could partly explain discrepancies between studies conducted in different populations. This heterogeneity may be attributable as well to differences in study design, genotyping methods, and environmental factors among the included studies. Because of the limited number of studies, the sources of heterogeneity could not be explored further. Third, subgroup analyses and formal assessment of publication bias could not be conducted because of the limited number of eligible studies. Although Funnel plot analyses were performed for comparisons including at least three studies, their interpretability and reliability remain limited due to the small number of studies available for each analysis. This is particularly relevant, as publication bias is often more noticeable in meta-analyses based on few and relatively small studies. In this study, publication bias may have resulted from several factors, including the limited availability of evidence due to the relatively recent emergence of research on lncRNA SNPs, insufficient reporting of data in otherwise eligible studies that precluded inclusion in quantitative synthesis, and the potential presence of selective or biased findings within the included studies. Additionally, only articles published in English were considered, which may have further contributed to publication bias. Therefore, these findings should be interpreted with caution. Future studies should include larger, well-designed cohorts from diverse populations, ideally incorporating data from high-volume centers, which may provide more robust and generalizable results. Further investigations should also explore potential gene-gene and gene-environment interactions. Further functional studies are also needed to clarify the biological mechanisms underlying the observed associations. In addition, advances in computational and data-driven approaches, including artificial intelligence-based methods, may facilitate the integration of genetic and clinical data to support precision oncology in GI cancers [77]. A clearer understanding of the functional consequences of these polymorphisms may further support their potential application as biomarkers or therapeutic targets in digestive system cancers.

## 5. Conclusions

Taken together, the present findings refine the current understanding of lncRNA genetics in digestive system cancers. Rather than supporting a generalized role for all lncRNA polymorphisms, the results indicate that MEG3 rs7158663 in CRC, H19 rs3024270 in HCC, PRNCR1 rs16901946 in GC and GAS5 rs145204276 in GC emerge as the most credible susceptibility candidates, whereas evidence for H19 rs2839698 and MALAT1 rs619586 in HCC and GAS5 rs145204276 in CRC remains limited. A major strength of this study is the systematic evaluation of recurrently investigated lncRNS SNPs using predefined eligibility criteria and comparable SNP-cancer associations. However, the findings should be interpreted with caution, as several analyses were based on a limited number of eligible studies. The distinction of this study may help inform future research by highlighting variants that may deserve further functional study and could have potential relevance for biomarker investigation or genetic risk stratification. Importantly, by systematically evaluating recurrently studied lncRNA polymorphisms across major digestive system cancers, the present study offers an updated summary of the available evidence and helps differentiate more consistent associations from those that remain uncertain. Future studies should focus on larger and ethnically diverse cohorts, as well as functional validation of the identified variants to clarify their biological relevance.

## Figures and Tables

**Table 1:** Included studies for meta-analyses. ^1^: Two-stage studies counted together. ^2^: Studies conducted in different populations. ^3^: Excluded from meta-analyses. ^4^: Data recalculated from Table 3 of the original study (Petkevicius et al. [35]). SNP: single nucleotide polymorphism. CRC: colorectal cancer. GC: gastric cancer. HCC: hepatocellular cancer. GAS5: Growth Arrest Specific 5. PRNCR1: prostate cancer-associated non-coding RNA1. HOTAIR: HOX transcript antisense RNA. MALAT1: Metastasis Associated Lung Adenocarcinoma Transcript 1. MEG3: Maternally Expressed 3. NA: not available.

First Author	Publishing Year	Cancer Type	lncRNA	SNP	Ethnicity	Number of Cases	Number of Controls	Total Sample Size	Genotype Distribution (n)						HWE Calculated	Genotyping Method and Sample Type	Reference
									Homozygous Mutant		Heterozygous Mutant		Wild Type				
									Case	Control	Case	Control	Case	Control			
Zhu Z	2016	CRC	GAS5	rs145204276	Chinese	813	926	1739	109	73	387	409	317	444	0.1116	targeted (qPCR (SYBR)); venous blood	[36]
Mirea CS	2025	CRC	GAS5	rs145204276	Romanian	156	195	351	2	1	39	27	115	167	0.9352	targeted (TaqMan real-time PCR); peripherial blood	[37]
Zheng Y ^1^	2016	CRC	GAS5	rs145204276	Chinese	1400	1400	2800	112	151	550	610	738	639	0.7633	targeted (PCR); blood and tumor tissues	[38]
Aminian K	2019	GC	GAS5	rs145204276	Iranian	130	230	360	6	20	36	84	88	126	0.2710	targeted (PCR); tissues	[39]
Li Q	2018	GC	GAS5	rs145204276	Chinese	853	954	1807	461	454	334	415	58	85	0.4759	targeted (RT-PCR); peripherial blood	[40]
Li Q ^2^	2018	GC	GAS5	rs145204276	Chinese	1253	1354	2607	682	638	483	593	88	123	0.3757	targeted (TaqMan real-time PCR); peripherial blood	[41]
Li L	2016	GC	PRNCR1	rs16901946	Chinese	219	394	613	2	29	92	135	125	230	0.1439	targeted (PCR-RFLP); NA	[42]
Hong JH	2019	GC	PRNCR1	rs16901946	Korean	437	357	794	38	32	191	147	208	178	0.8343	targeted (TaqMan real-time PCR); peripherial blood	[43]
He BS	2017	GC	PRNCR1	rs16901946	Chinese	494	494	988	30	17	203	176	261	301	0.1529	targeted (Sequenom MassARRAY); peripherial blood	[44]
Petkevicius V ^3^	2020	GC	H19	rs217727	Lithuanian, Latvian, German	610 ^4^	473 ^4^	1089	29	30	229	184	352	259	0.7245	targeted (TaqMan real-time PCR); peripherial blood	[35]
Yang C ^3^	2015	GC	H19	rs217727	Chinese Han	500	500	1000	88	63	252	244	160	193	0.2957	targeted (TaqMan real-time PCR); serum	[45]
Wei M ^3^	2019	GC	H19	rs217727	Chinese Han	225	200	425	65	93	72	44	88	63	<0.0001	targeted (TaqMan real-time PCR); peripherial blood	[46]
Tan T	2021	HCC	H19	rs2839698	Chinese Han	213	957	1170	33	94	78	424	102	439	0.5679	targeted (TaqMan real-time PCR); peripherial blood	[47]
Wu ER	2019	HCC	H19	rs2839698	Chinese	359	1190	1549	140	532	178	524	41	134	0.7718	targeted (TaqMan real-time PCR); tissues	[48]
Yang ML	2018	HCC	H19	rs2839698	Chinese	466	462	928	40	32	211	185	215	245	0.297	targeted (KASP); NA	[49]
Tan T	2021	HCC	H19	rs3024270	Chinese Han	213	957	1170	76	204	87	489	50	264	0.4216	targeted (TaqMan real-time PCR); peripherial blood	[47]
Wu ER	2019	HCC	H19	rs3024270	Chinese	359	1190	1549	85	263	187	593	87	334	0.9945	targeted (TaqMan real-time PCR); tissues	[48]
Yang ML	2018	HCC	H19	rs3024270	Chinese	471	466	937	95	81	225	215	151	170	0.3409	targeted (KASP); NA	[49]
Pan W	2016	GC	HOTAIR	rs4759314	Chinese	500	1000	1500	1	3	48	83	451	914	0.4482	targeted (PCR-RFLP); peripherial blood	[50]
Abdi E ^3^	2020	GC	HOTAIR	rs4759314	Iranian	300	300	600	0	0	14	10	286	290	0.7691	targeted (microarray); peripherial blood	[51]
Guo W	2015	GC	HOTAIR	rs4759314	Chinese	515	654	1169	1	1	53	64	461	589	0.5872	targeted (PCR-RFLP); peripherial blood	[52]
Du M	2015	GC	HOTAIR	rs4759314	Chinese	1275	1644	2919	6	8	186	172	1083	1464	0.2297	targeted (TaqMan real-time PCR); peripherial blood	[53]
Motawi TMK ^3^	2019	HCC	MALAT1	rs619586	Egyptian	70	70	140	4	7	16	17	50	46	0.0134	targeted (TaqMan real-time PCR); peripherial blood	[54]
Ji X	2019	HCC	MALAT1	rs619586	Chinese Han	624	618	1242	7	5	93	82	522	531	0.3574	targeted (TaqMan real-time PCR); peripherial blood	[55]
Yuan LT	2019	HCC	MALAT1	rs619586	Chinese Han	394	1199	1593	3	10	61	175	330	1014	0.4232	targeted (TaqMan real-time PCR); peripherial blood	[56]
Wang B	2018	HCC	MALAT1	rs619586	Chinese Han	518	806	1324	1	9	83	113	434	684	0.0800	targeted (KASP); peripherial blood	[57]
Liu Y	2012	HCC	MALAT1	rs619586	Chinese Han	1268	1330	2598	5	10	169	205	1094	1115	0.8600	targeted (TaqMan real-time PCR); venous blood	[58]
Gao X	2021	CRC	MEG3	rs7158663	Chinese Han	430	445	875	43	30	185	159	202	256	0.4349	Direct sequencing, targeted (qPCR (SYBR)); peripherial blood	[59]
Elhelaly Elsherbeny M	2023	CRC	MEG3	rs7158663	Egyptian	160	160	320	32	8	50	56	78	96	0.9638	targeted (TaqMan real-time PCR); serum	[60]
Cao X	2016	CRC	MEG3	rs7158663	Chinese	516	517	1033	52	31	200	188	264	298	0.8520	targeted (TaqMan real-time PCR); peripherial blood	[61]

## Data Availability

The original contributions presented in this study are included in the article and in the Supplementary Materials. Further inquiries can be directed to the corresponding authors.

## Supplementary Materials

The following supporting information can be downloaded at: https://www.mdpi.com/article/10.3390/cancers18121916/s1, Figure S1: Detailed search strategy. Figure S2: Funnel plots for visual assessment of potential publication bias in meta-analyses including at least three studies. Figure S3: Forest plots of the association between GAS5 rs145204276 polymorphism and CRC risk under different genetic models: (a) allelic model; (b) dominant model; (c) recessive model. Table S1: PRISMA 2020 main checklist. Table S2: PRISMA 2020 checklists for abstracts. Table S3: Newcastle-Ottawa Scale quality assessment of included studies.

## Abbreviations

The following abbreviations are used in this manuscript:

GI	Gastrointestinal
SNP	Single nucleotide polymorphism
lncRNA	Long non-coding RNA
CRC	Colorectal cancer
GC	Gastric cancer
HCC	Hepatocellular cancer
HWE	Hardy–Weinberg equilibrium
NOS	Newcastle-Ottawa Scale
