# Supplementary material for: The Role of lncRNA Polymorphisms in Digestive System Cancers: A Systematic Review and Meta-Analysis
Source: Cancers (Basel). 2026 Jun 12;18(12):1916. doi: 10.3390/cancers18121916 (PMC13297490; doi:10.3390/cancers18121916)
Supplement: Supplementary file 1 [file cancers-18-01916-s001.zip › Supplementary Figure S1.pdf]

Supplementary Figure S1. Detailed search strategy

1. **Ovid/MEDLINE**

Ovid MEDLINE(R) ALL <1946 to May 08, 2026>

1 exp Digestive System Neoplasms/

2 ((gastrointestinal or gastro-intestinal or esophageal or oesophageal or pancrea* or gastric or stomach or intestin* or duodenal or ileal or jejunal or cecal or appendiceal or colorectal or colon* or sigmoid or rectal or anus or "anal gland" or hepatocellular or liver or "biliary tract" or "bile duct" or gallbladder) adj3 (neoplasm* or cancer or tumor* or tumour* or carcinoma or malignan* or metastas*)).mp.

3 1 or 2

4 RNA, Long Noncoding/

5 ((long or large) adj3 ("non-coding RNA*" or "noncoding RNA*" or "ncRNA*" or "untranslated RNA*" or "non protein coding RNA*")).mp.

6 (lncRNA* or "lnc RNA*" or "linc RNA*").mp.

7 4 or 5 or 6

8 Polymorphism, Single Nucleotide/

9 Genetic Predisposition to Disease/

10 (polymorphism or variation* or variant* or mutation* or "SNP*").mp.

11 8 or 9 or 10

12 3 and 7 and 11

:1098

2. **Scopus**

(TITLE-ABS-KEY ((gastrointestinal OR gastro-intestinal OR esophageal OR oesophageal OR pancrea* OR gastric OR stomach OR intestin* OR duodenal OR ileal OR jejunal OR cecal OR appendiceal OR colorectal OR colon* OR sigmoid OR rectal OR anus OR "anal gland" OR hepatocellular OR liver OR "biliary tract" OR "bile duct" OR gallbladder) W/3 (neoplasm* OR cancer OR tumor* OR tumour* OR carcinoma OR malignan* OR metastas*))) AND ((TITLE-ABS-KEY (((long OR large) W/3 ("non-coding rna*" OR "noncoding rna*" OR "ncrna*" OR "untranslated rna*" OR "non protein coding rna*")))) OR (TITLE-ABS-KEY (lncrna* OR "lnc rna*" OR "linc rna*"))) AND (TITLE-ABS-KEY (polymorphism OR variation* OR variant* OR mutation* OR "snp*"))

:2460

3. **Embase**

('digestive system tumor'/exp OR ((gastrointestinal OR 'gastro intestinal' OR esophageal OR oesophageal OR pancrea* OR gastric OR stomach OR intestin* OR duodenal OR ileal OR jejunal OR cecal OR appendiceal OR colorectal OR colon* OR sigmoid OR rectal OR anus OR 'anal gland' OR hepatocellular OR liver OR 'biliary tract' OR 'bile duct' OR gallbladder) NEAR/3 (neoplasm* OR cancer OR tumor* OR tumour* OR carcinoma OR malignan* OR metastas*))) AND ('long untranslated rna'/exp OR ((long OR large) NEAR/3 ('non-coding rna*' OR 'noncoding rna*' OR ncrna* OR 'untranslated rna*' OR 'non protein coding rna*')) OR lncrna* OR 'lnc rna*' OR 'linc rna*') AND ('single nucleotide polymorphism'/exp OR polymorphism OR variation* OR variant* OR mutation* OR 'snp*')

:2710

4. **Web of Science** (Core collection)

#1 TI=(((gastrointestinal OR "gastro intestinal" OR esophageal OR oesophageal OR pancrea* OR gastric OR stomach OR intestin* OR duodenal OR ileal OR jejunal OR cecal OR appendiceal OR colorectal OR colon* OR sigmoid OR rectal OR anus OR "anal gland" OR hepatocellular OR liver OR "biliary tract" OR "bile duct" OR gallbladder) NEAR/3 (neoplasm* OR cancer OR tumor* OR tumour* OR carcinoma OR malignan* OR metastas*)))

#2 AB=(((gastrointestinal OR "gastro intestinal" OR esophageal OR oesophageal OR pancrea* OR gastric OR stomach OR intestin* OR duodenal OR ileal OR jejunal OR cecal OR appendiceal OR colorectal OR colon* OR sigmoid OR rectal OR anus OR "anal gland" OR hepatocellular OR liver OR "biliary tract" OR "bile duct" OR gallbladder) NEAR/3 (neoplasm* OR cancer OR tumor* OR tumour* OR carcinoma OR malignan* OR metastas*)))

#3 #1 OR #2

#4 TI=((((long OR large) NEAR/3 ("non-coding rna*" OR "noncoding rna*" OR ncrna* OR "untranslated rna*" OR "non protein coding rna*")) OR (lncrna* OR "lnc rna*" OR "linc rna*")))

#5 AB=((((long OR large) NEAR/3 ("non-coding rna*" OR "noncoding rna*" OR ncrna* OR "untranslated rna*" OR "non protein coding rna*")) OR (lncrna* OR "lnc rna*" OR "linc rna*")))

#6 #4 OR #5

#7 TI=((polymorphism OR variation* OR variant* OR mutation* OR "snp*"))

#8 AB=((polymorphism OR variation* OR variant* OR mutation* OR "snp*"))

#9 #7 OR #8

#10 #3 AND #6 AND #9

:**876**
