# Supplementary material for: The Role of lncRNA Polymorphisms in Digestive System Cancers: A Systematic Review and Meta-Analysis
Source: Cancers (Basel). 2026 Jun 12;18(12):1916. doi: 10.3390/cancers18121916 (PMC13297490; doi:10.3390/cancers18121916)
Supplement: Supplementary file 1 [file cancers-18-01916-s001.zip › Supplementary Figure S2.pdf]

Supplementary Figure S2. Funnel plots for visual assessment of potential publication bias in meta-analyses including at least three studies

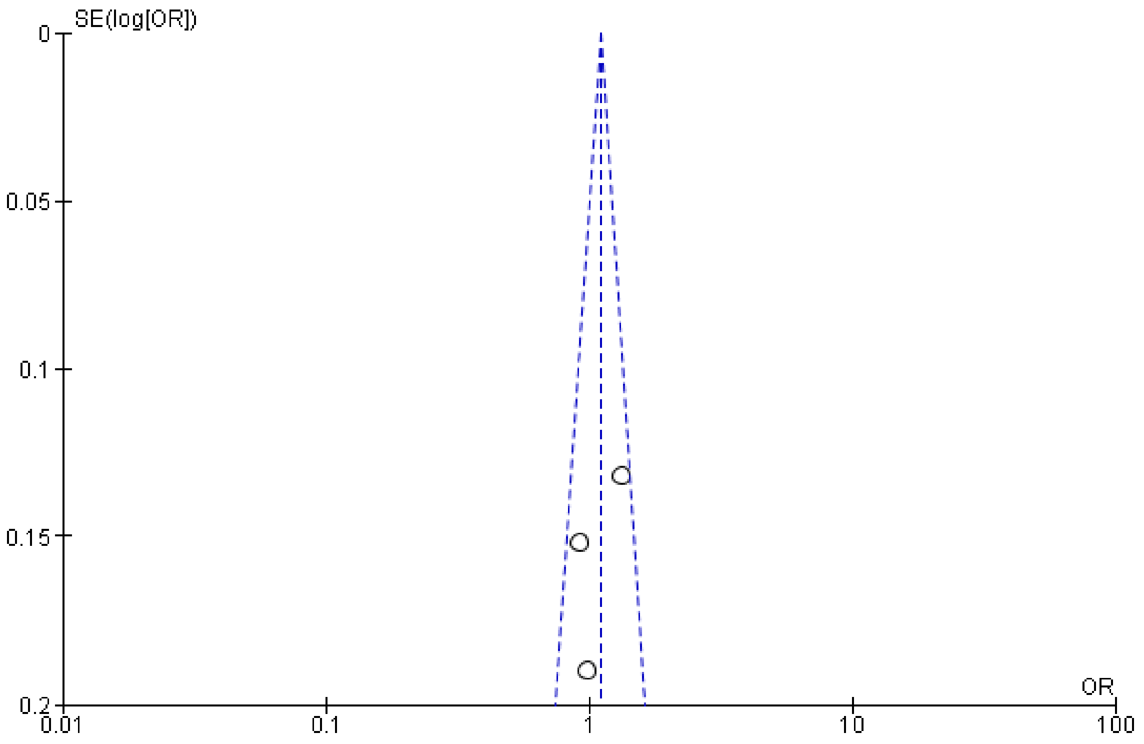

Funnel plot of studies of the H19 rs2839698 polymorphism and HCC risk under the dominant genetic model.

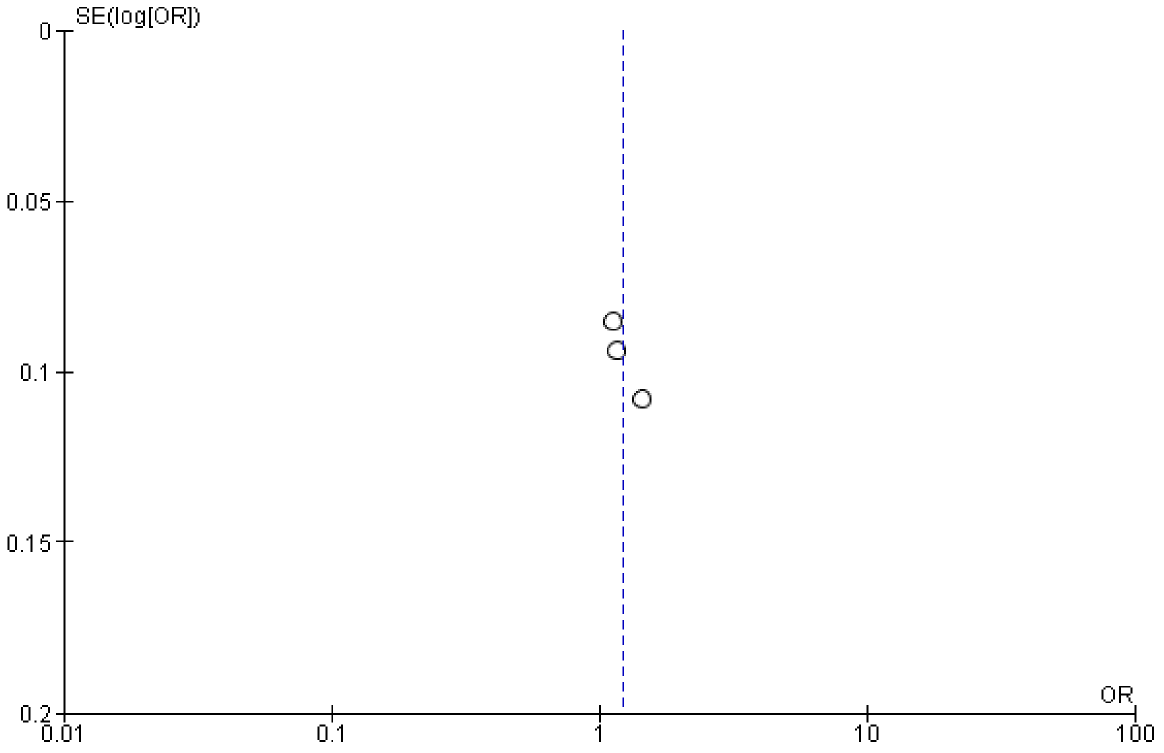

Funnel plot of studies of the H19 rs3024270 polymorphism and HCC risk under the allelic genetic model.

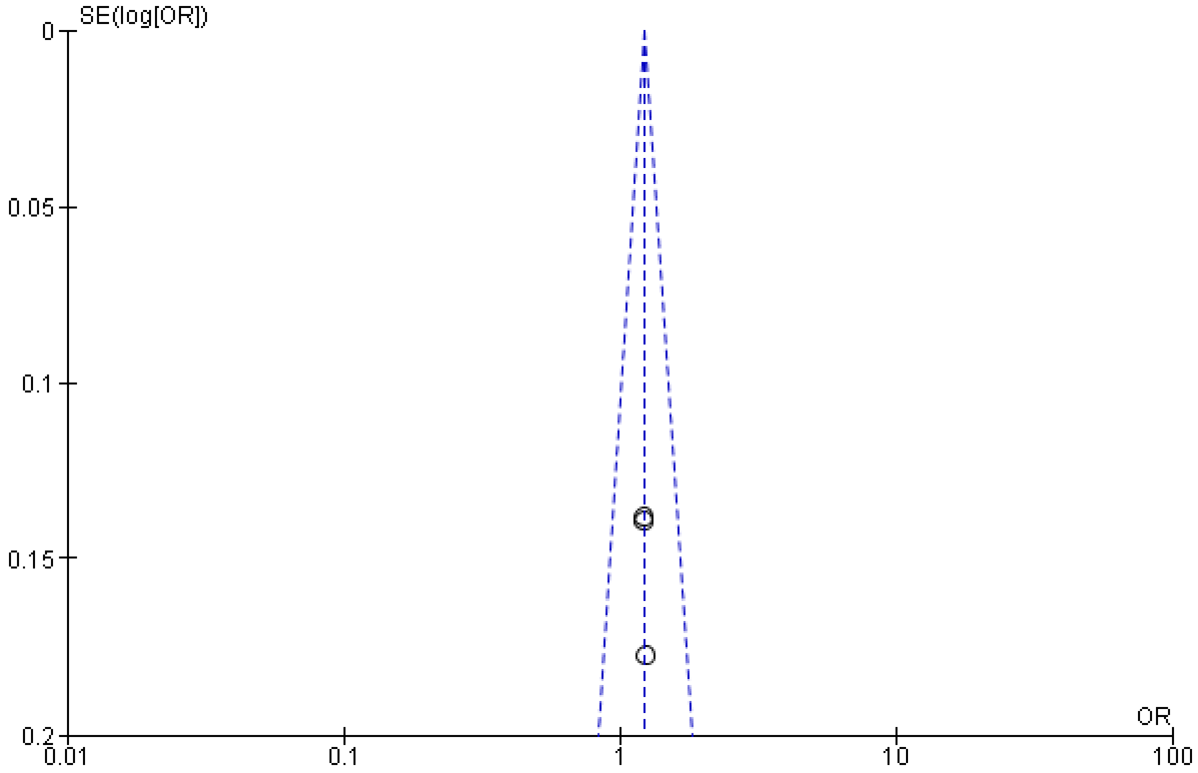

Funnel plot of studies of the H19 rs3024270 polymorphism and HCC risk under the dominant genetic model.

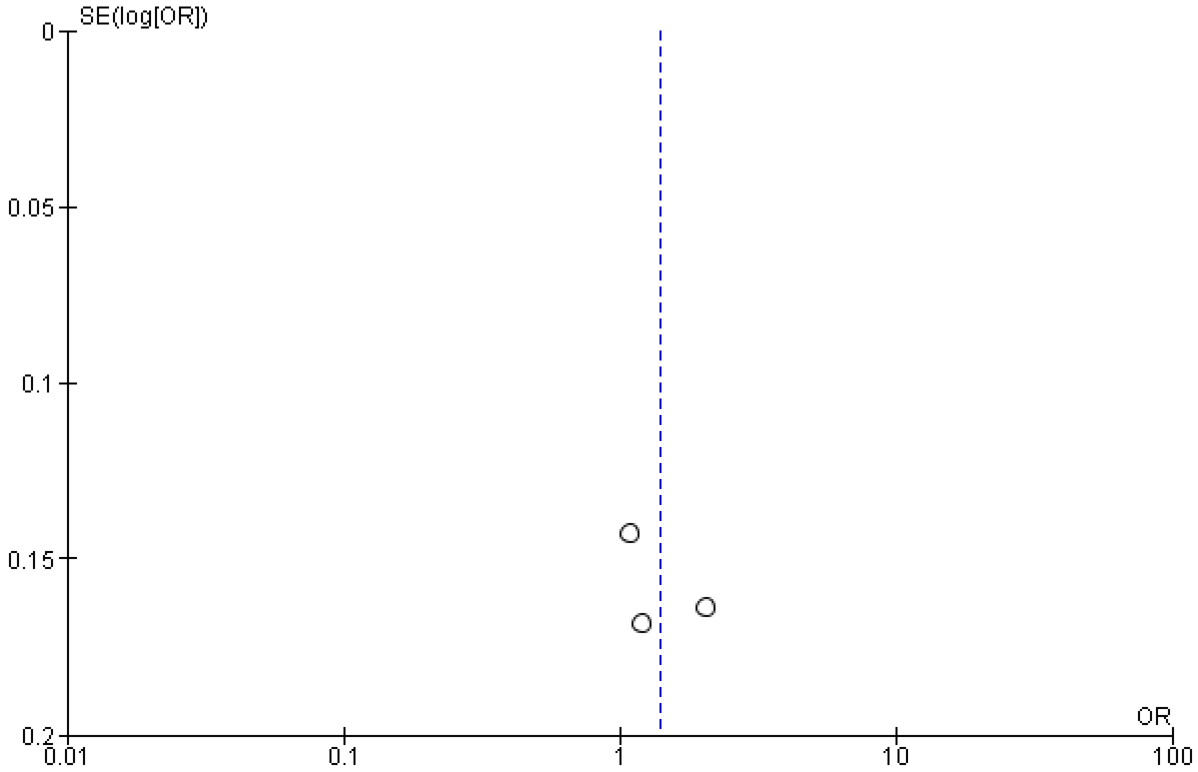

Funnel plot of studies of the H19 rs3024270 polymorphism and HCC risk under the recessive genetic model.

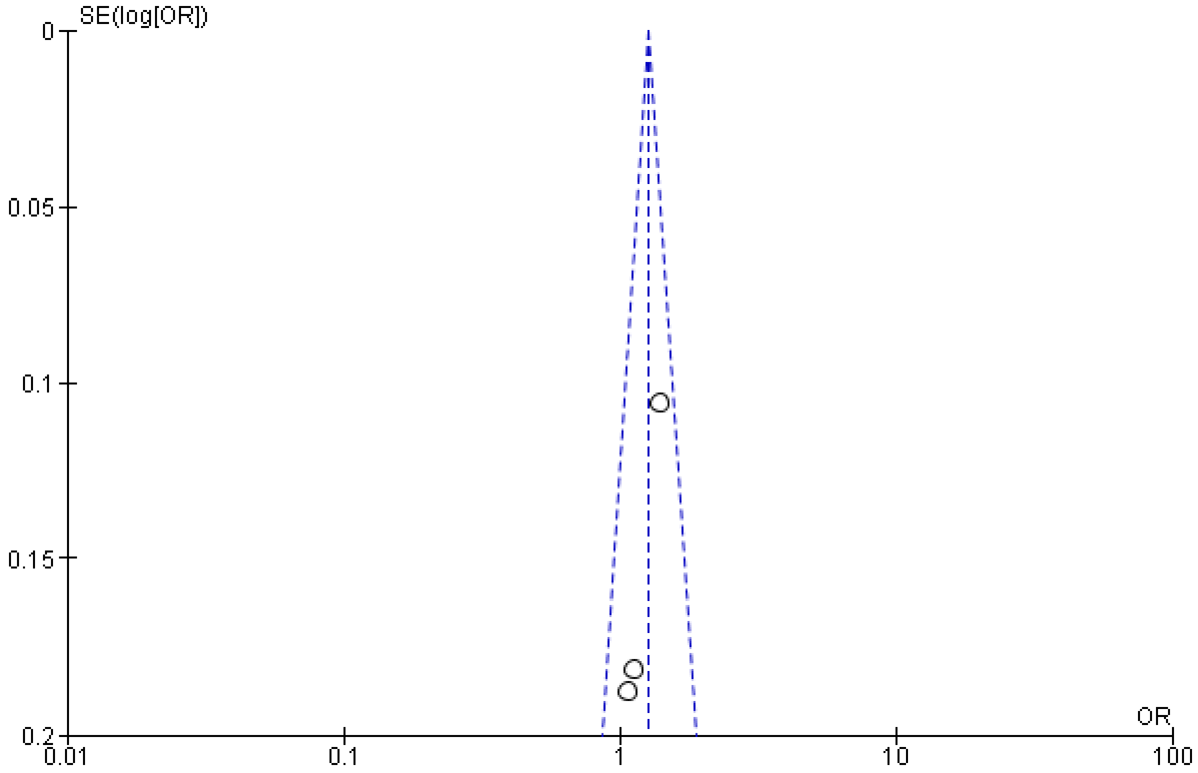

Funnel plot of studies of the HOTAIR rs4759314 polymorphism and GC risk under the allelic genetic model.

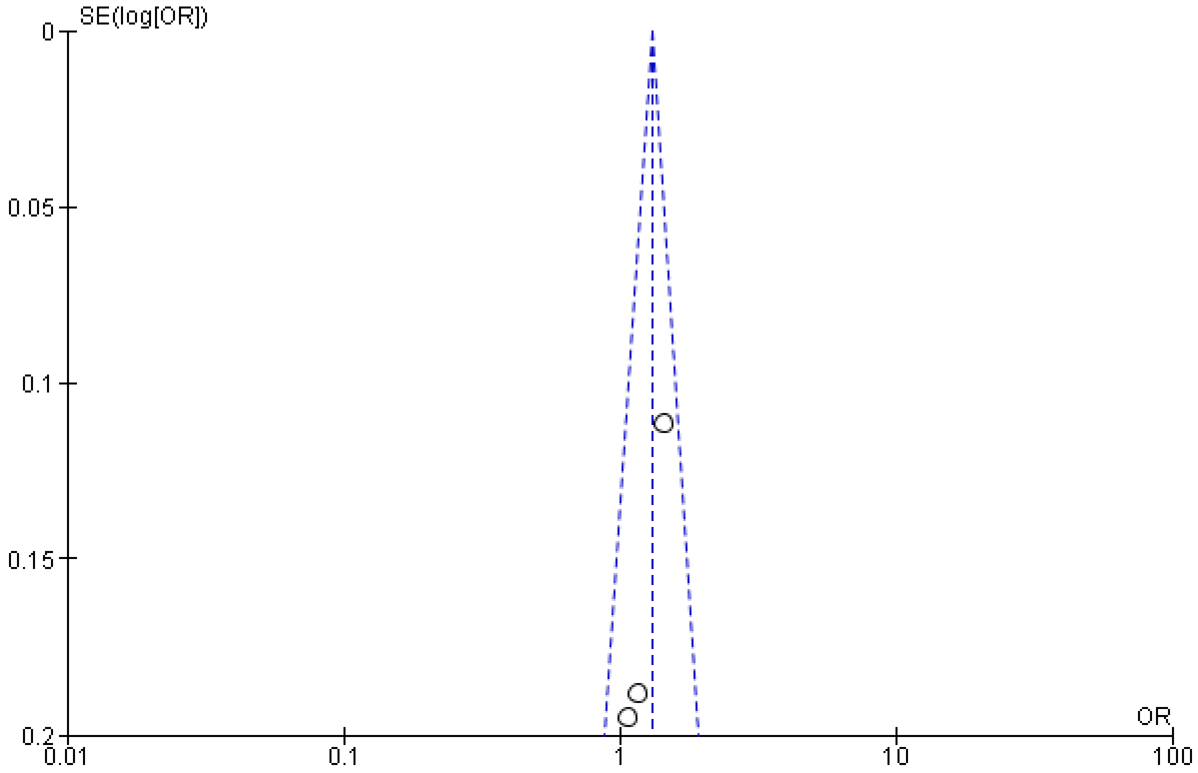

Funnel plot of studies of the HOTAIR rs4759314 polymorphism and GC risk under the dominant genetic model.

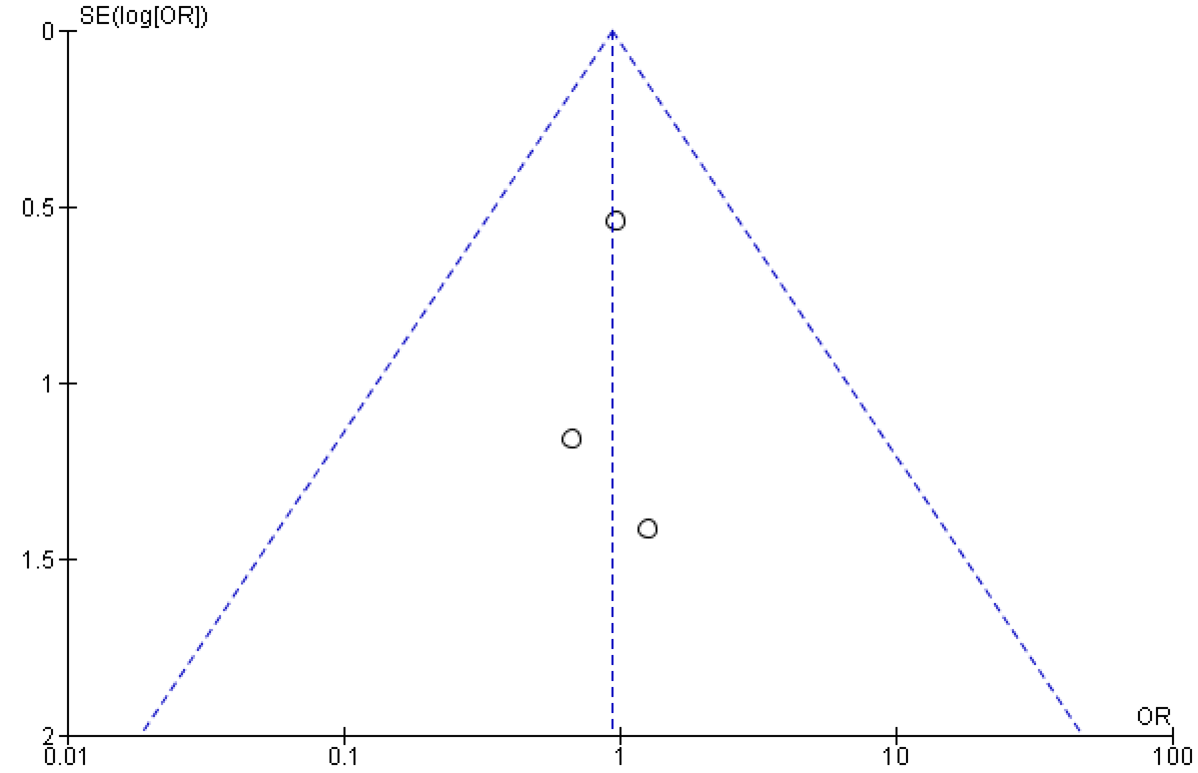

Funnel plot of studies of the HOTAIR rs4759314 polymorphism and GC risk under the recessive genetic model.

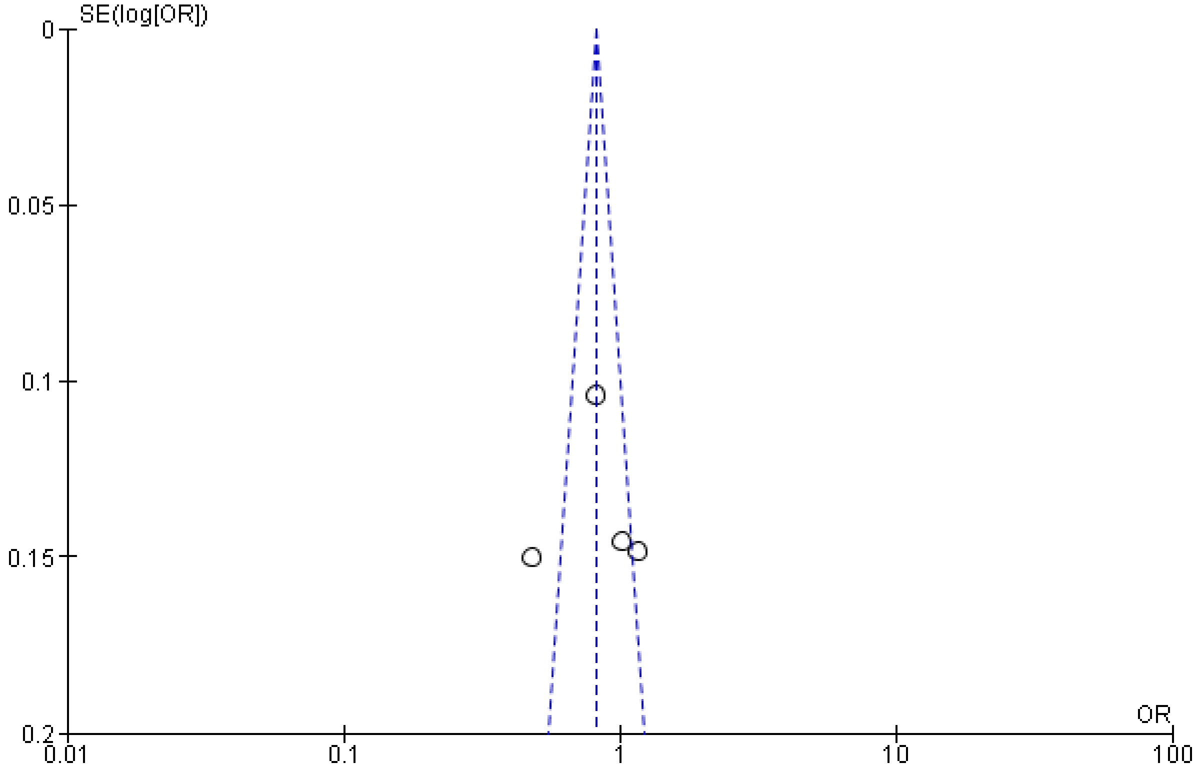

Funnel plot of studies of the MALAT1 rs619586 polymorphism and HCC risk under the allelic genetic model.

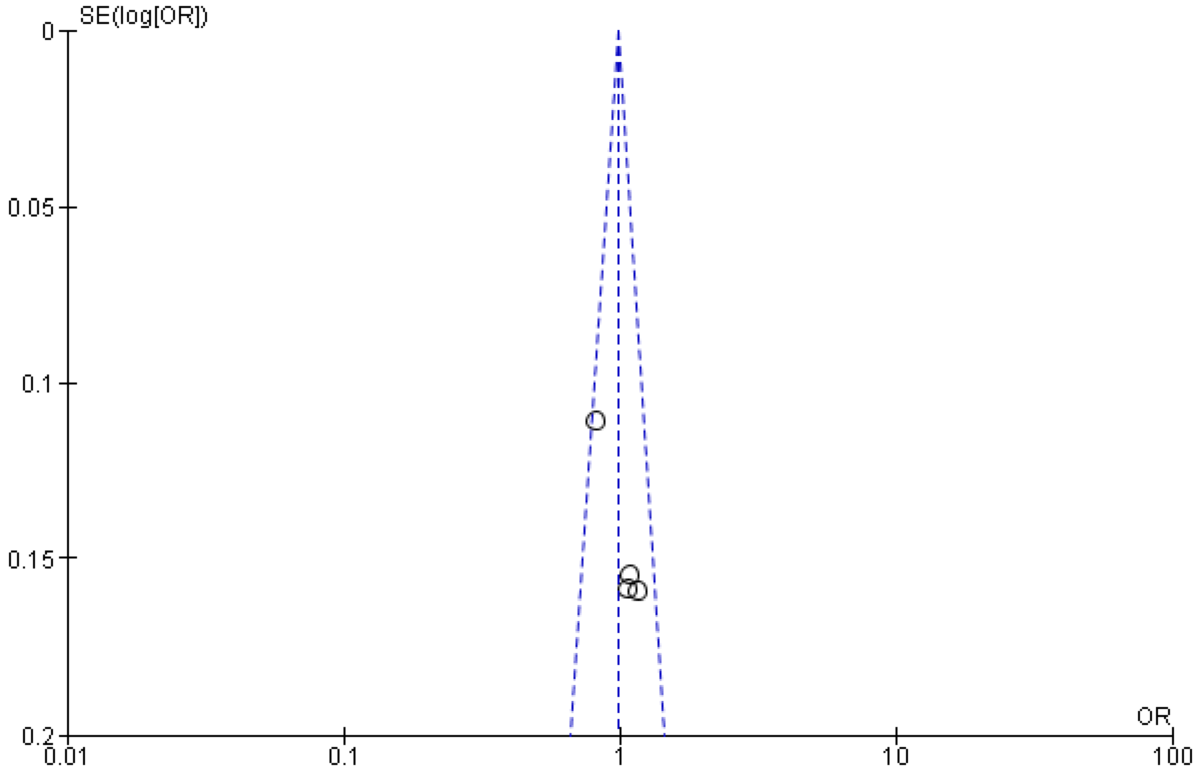

Funnel plot of studies of the MALAT1 rs619586 polymorphism and HCC risk under the dominant genetic model.

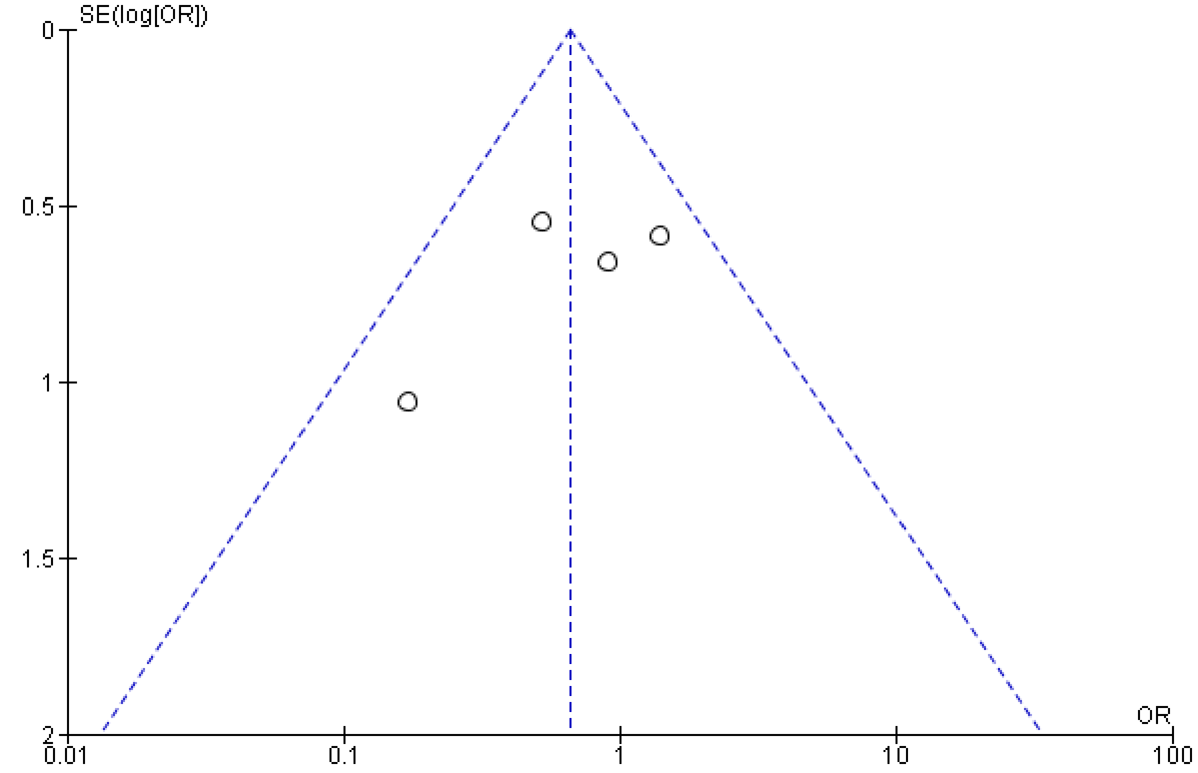

Funnel plot of studies of the MALAT1 rs619586 polymorphism and HCC risk under the recessive genetic model.

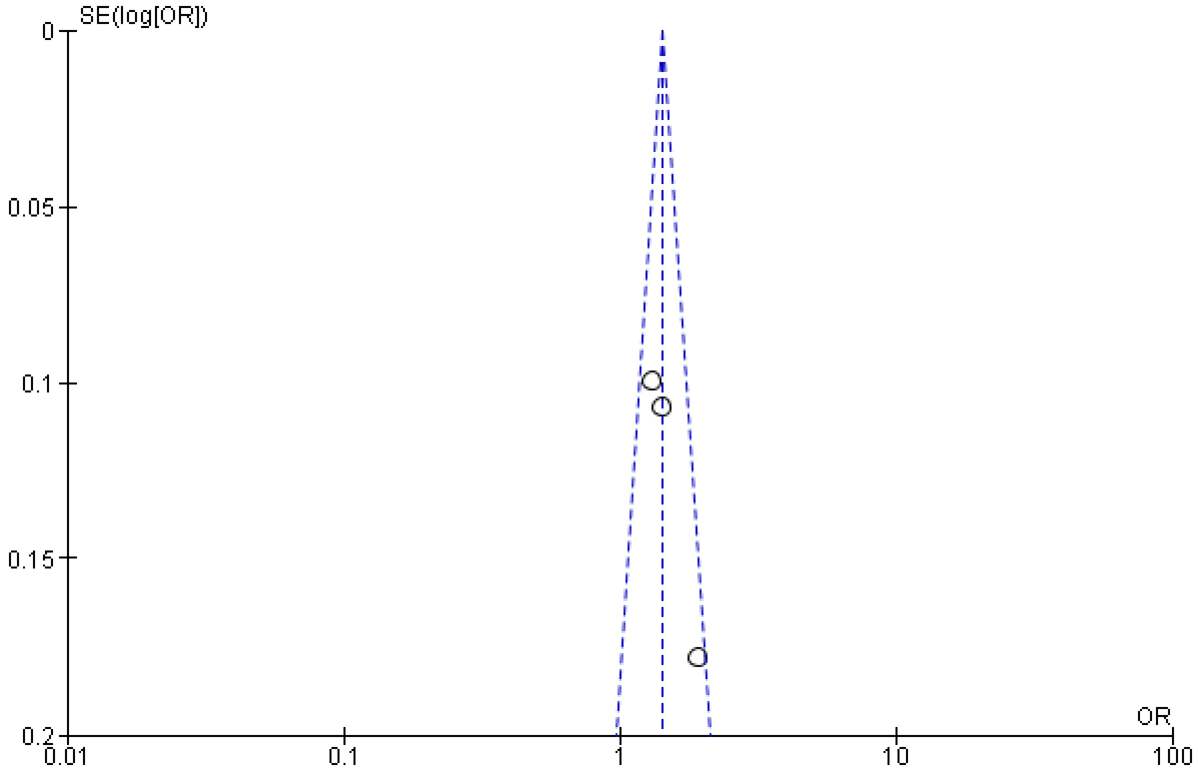

Funnel plot of studies of the MEG3 rs7158663 polymorphism and CRC risk under the allelic genetic model.

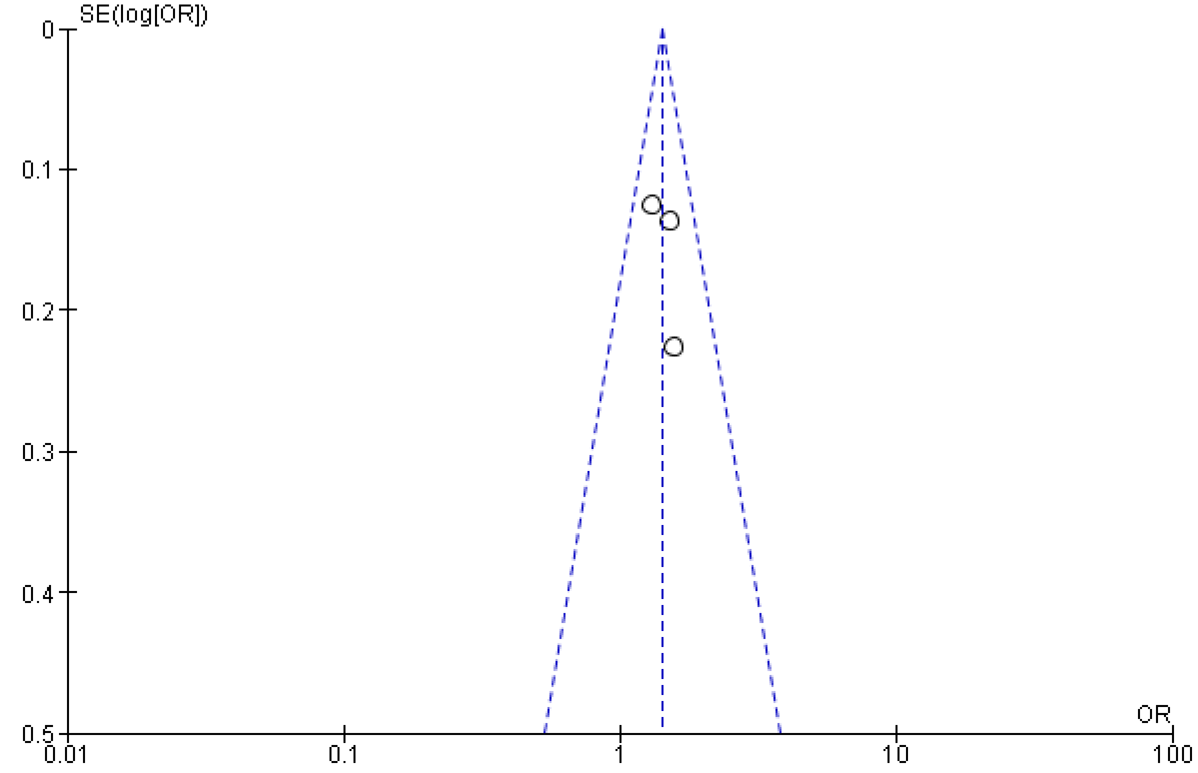

Funnel plot of studies of the MEG3 rs7158663 polymorphism and CRC risk under the dominant genetic model.

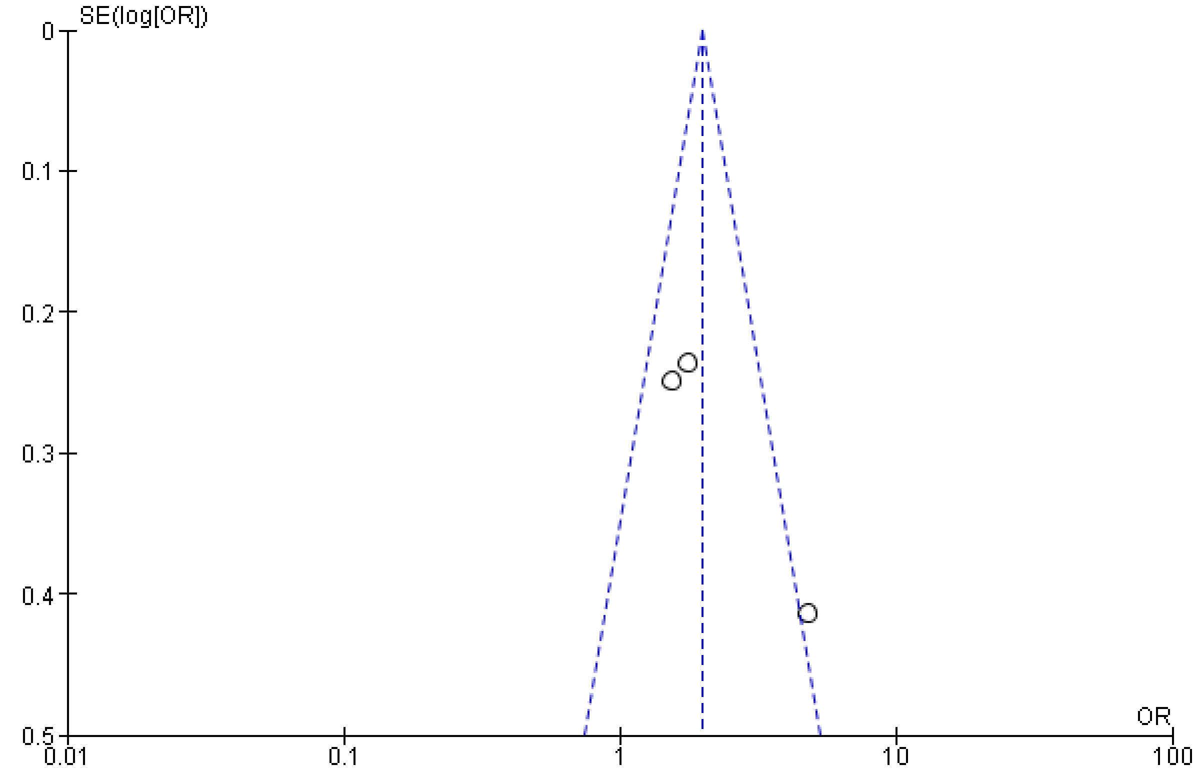

Funnel plot of studies of the MEG3 rs7158663 polymorphism and CRC risk under the recessive genetic model.

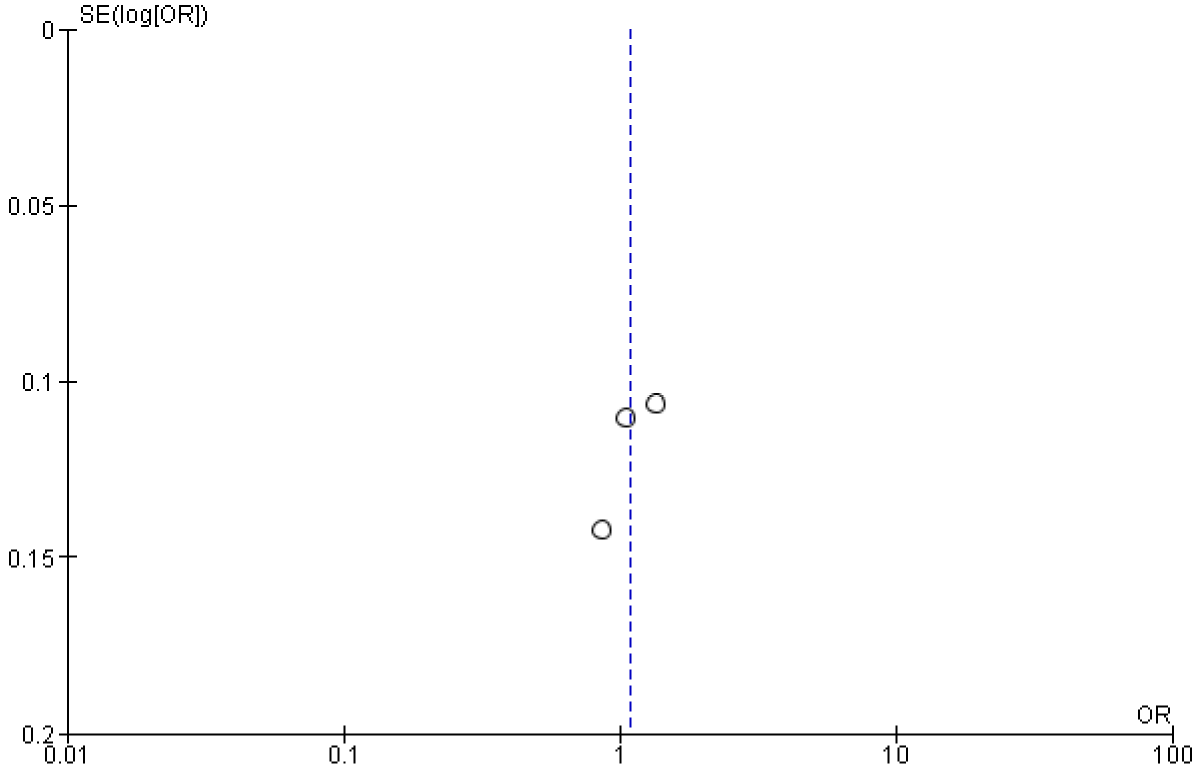

Funnel plot of studies of the PRNCR1 rs16901946 polymorphism and GC risk under the allelic genetic model.

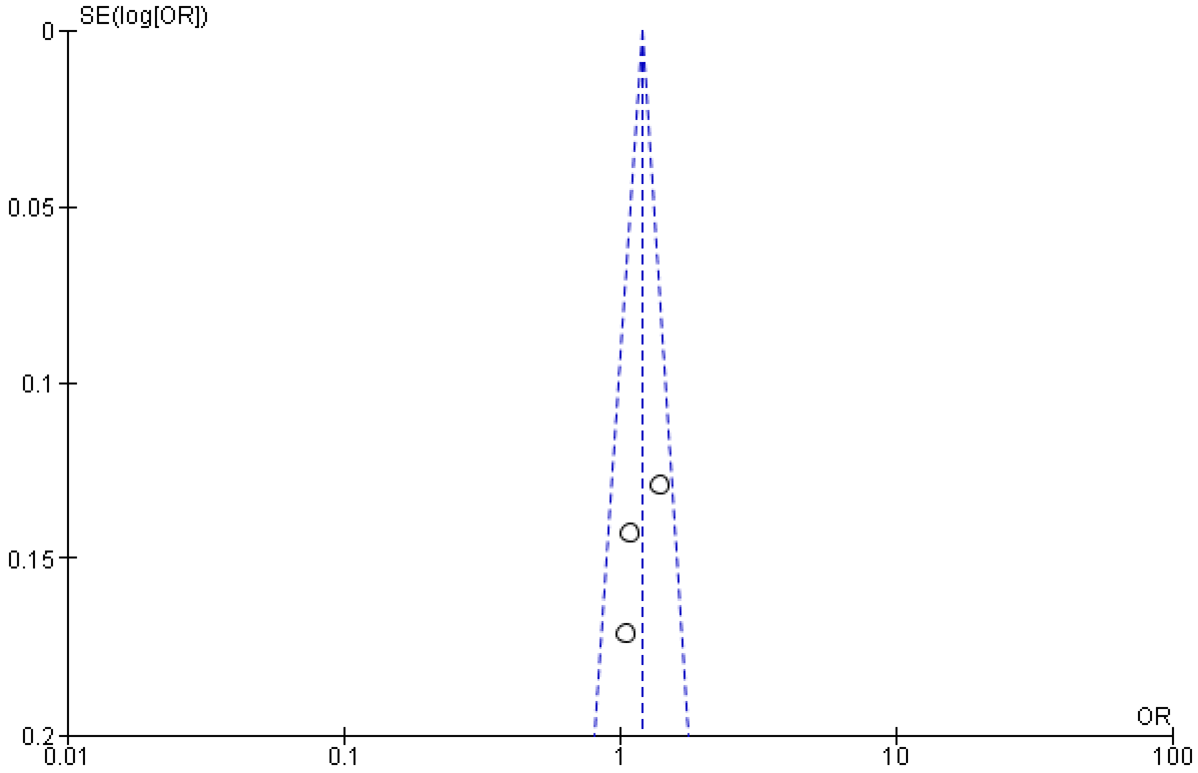

Funnel plot of studies of the PRNCR1 rs16901946 polymorphism and GC risk under the dominant genetic model.

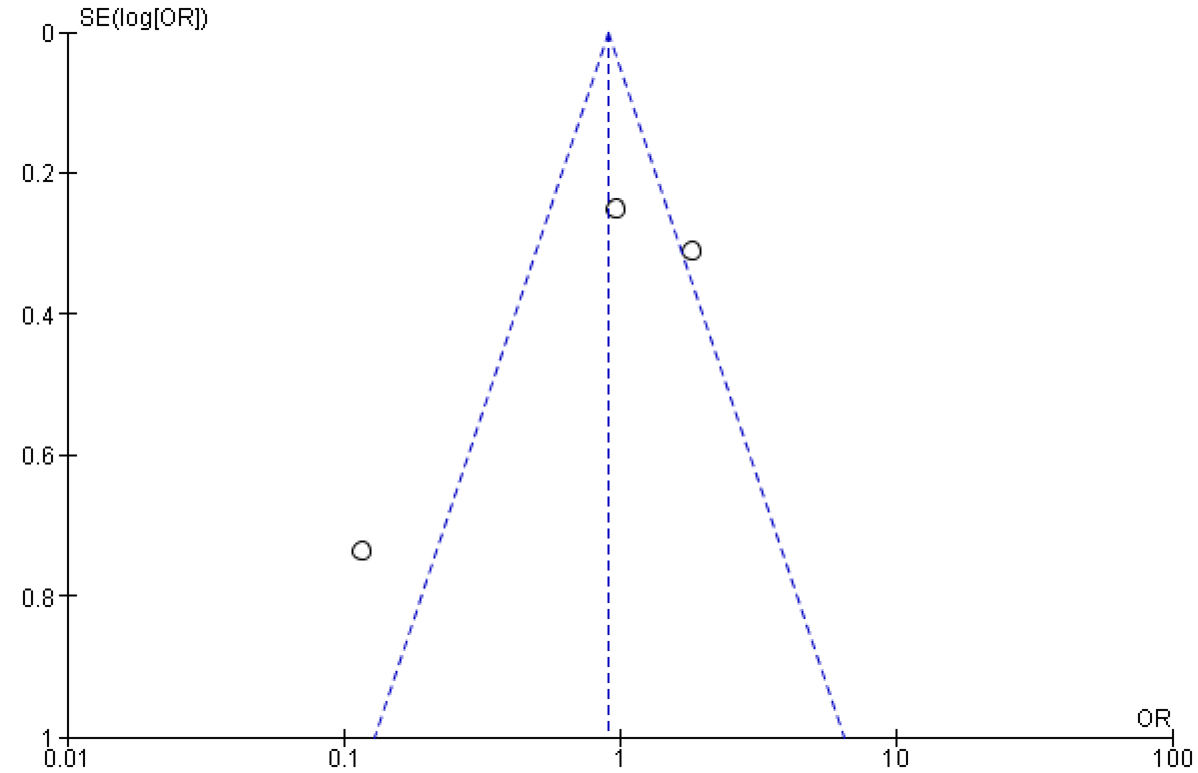

Funnel plot of studies of the PRNCR1 rs16901946 polymorphism and GC risk under the recessive genetic model.

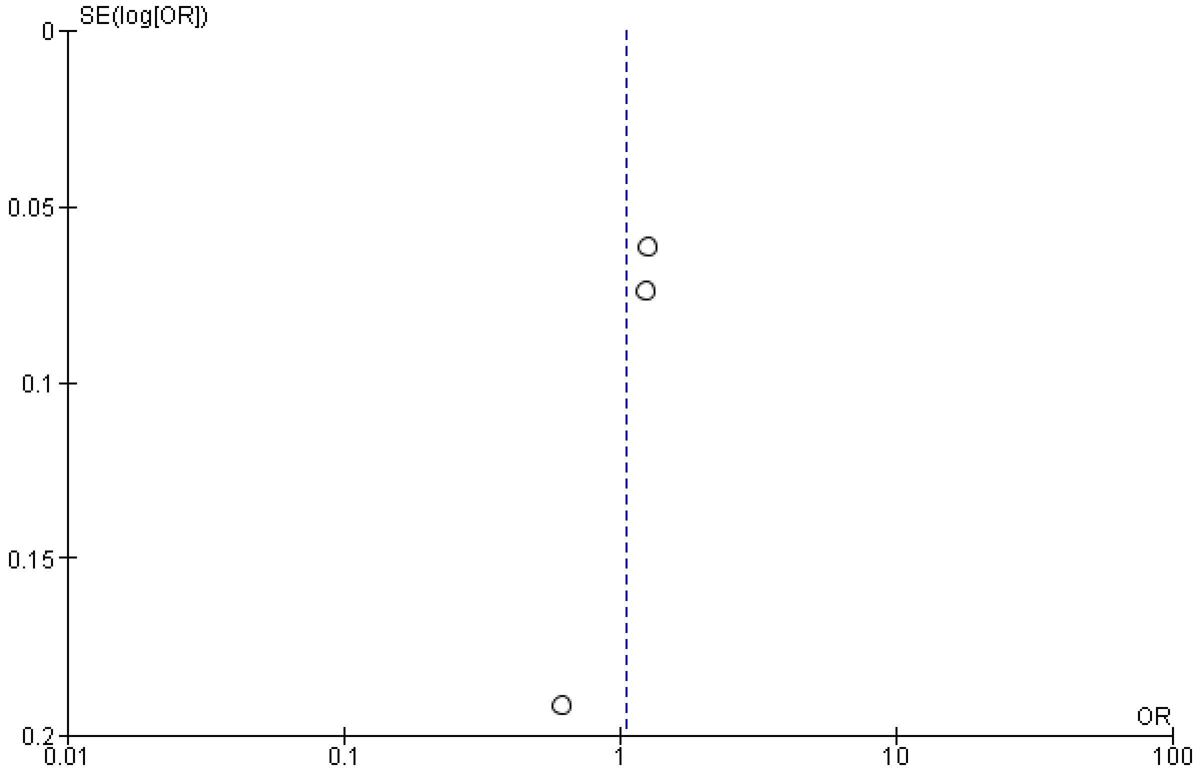

Funnel plot of studies of the GAS5 rs145204276 polymorphism and GC risk under the allelic genetic model.

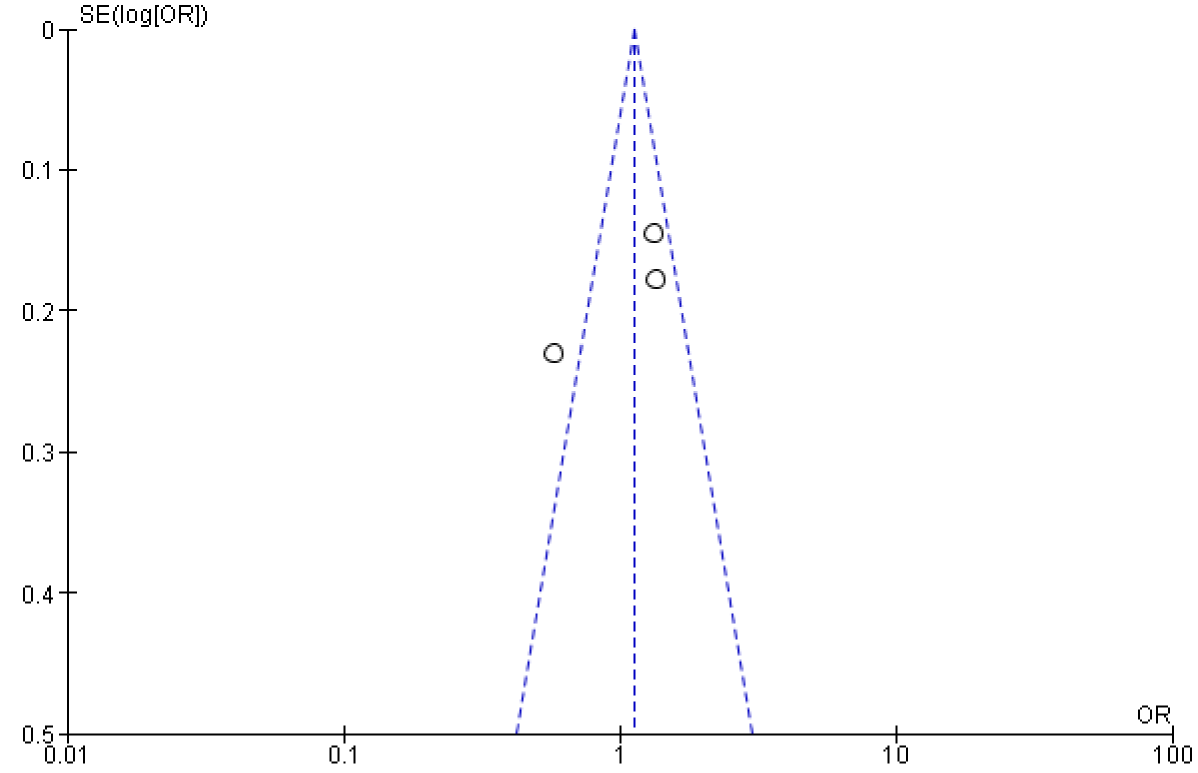

Funnel plot of studies of the GAS5 rs145204276 polymorphism and GC risk under the dominant genetic model.

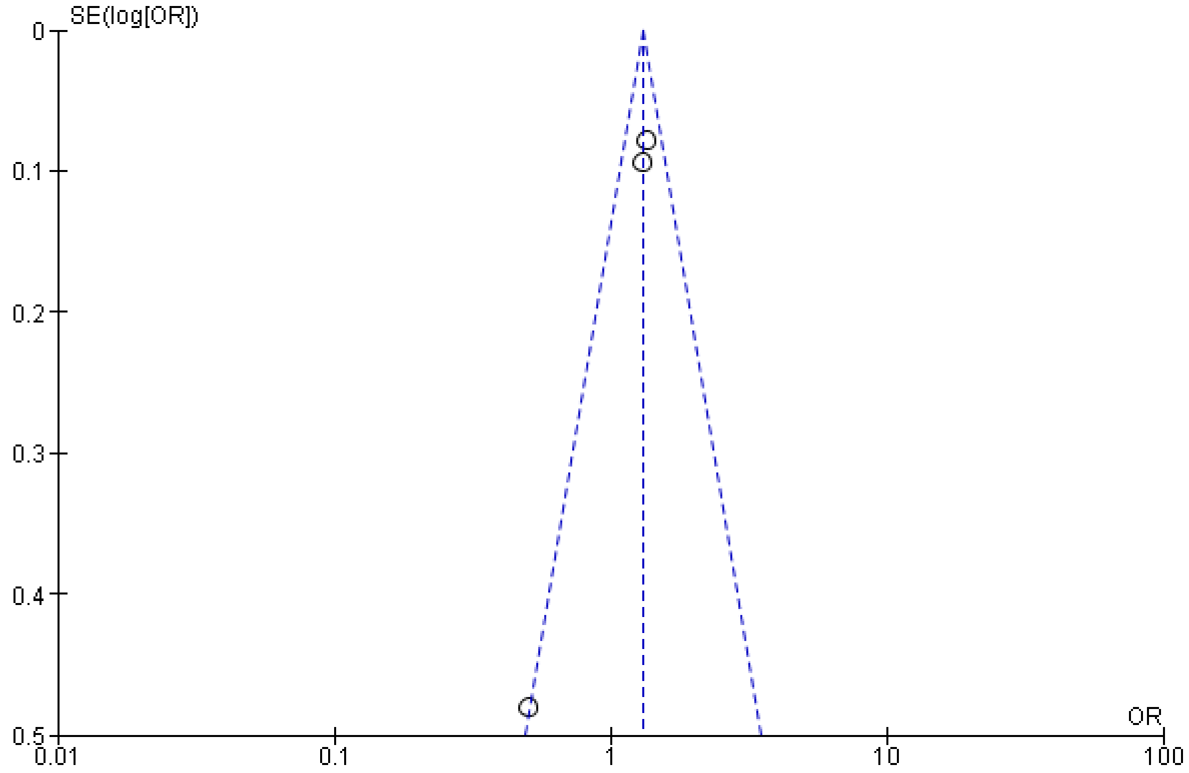

Funnel plot of studies of the GAS5 rs145204276 polymorphism and GC risk under the recessive genetic model.

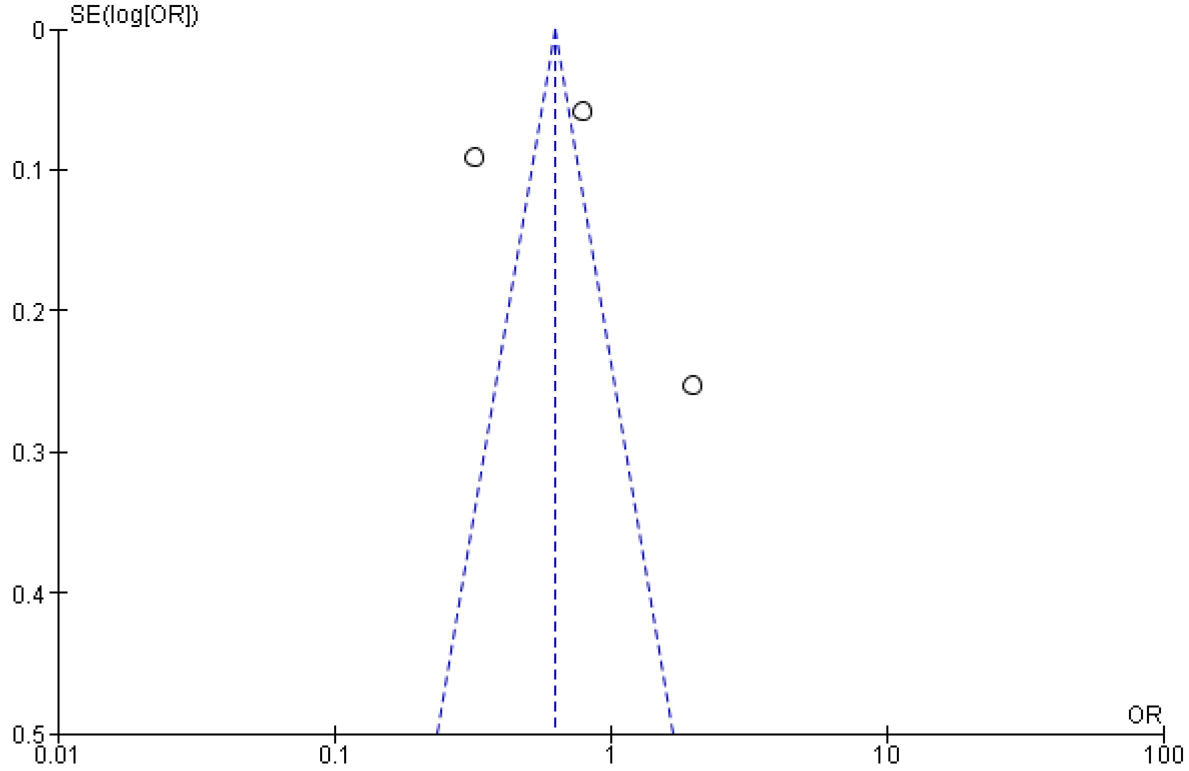

Funnel plot of studies of the GAS5 rs145204276 polymorphism and CRC risk under the allelic genetic model.

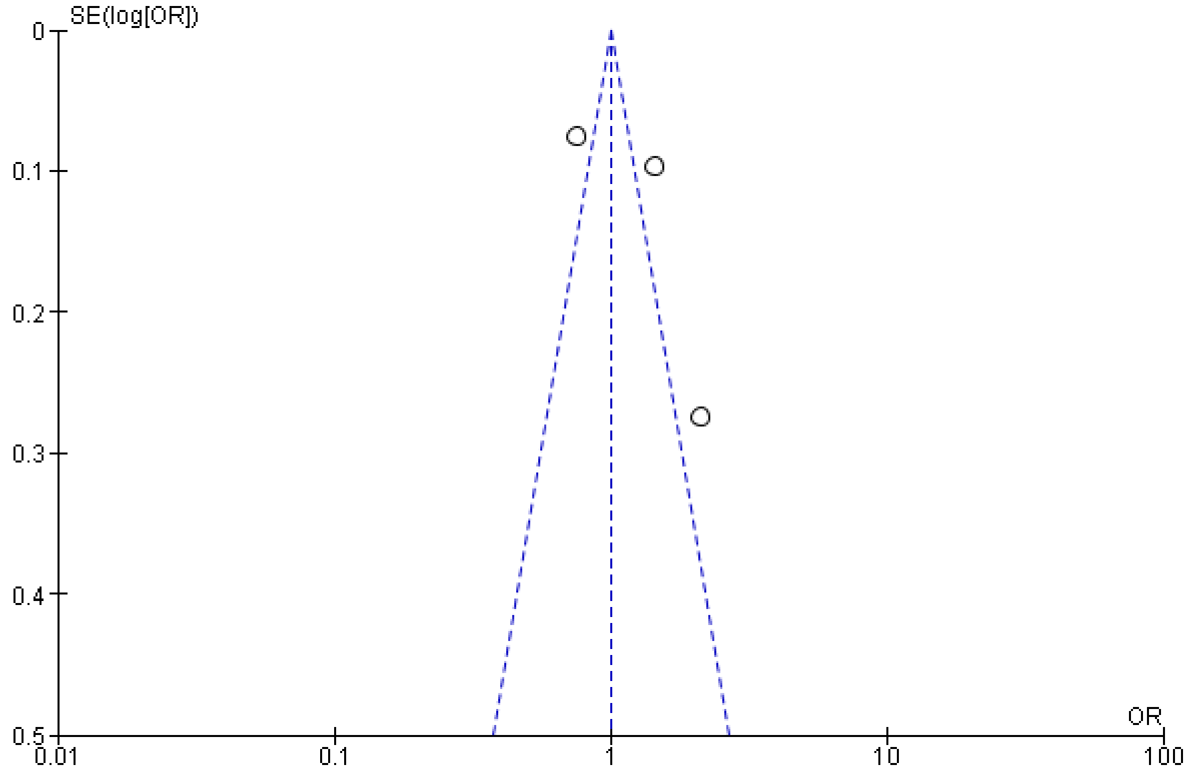

Funnel plot of studies of the GAS5 rs145204276 polymorphism and CRC risk under the dominant genetic model.

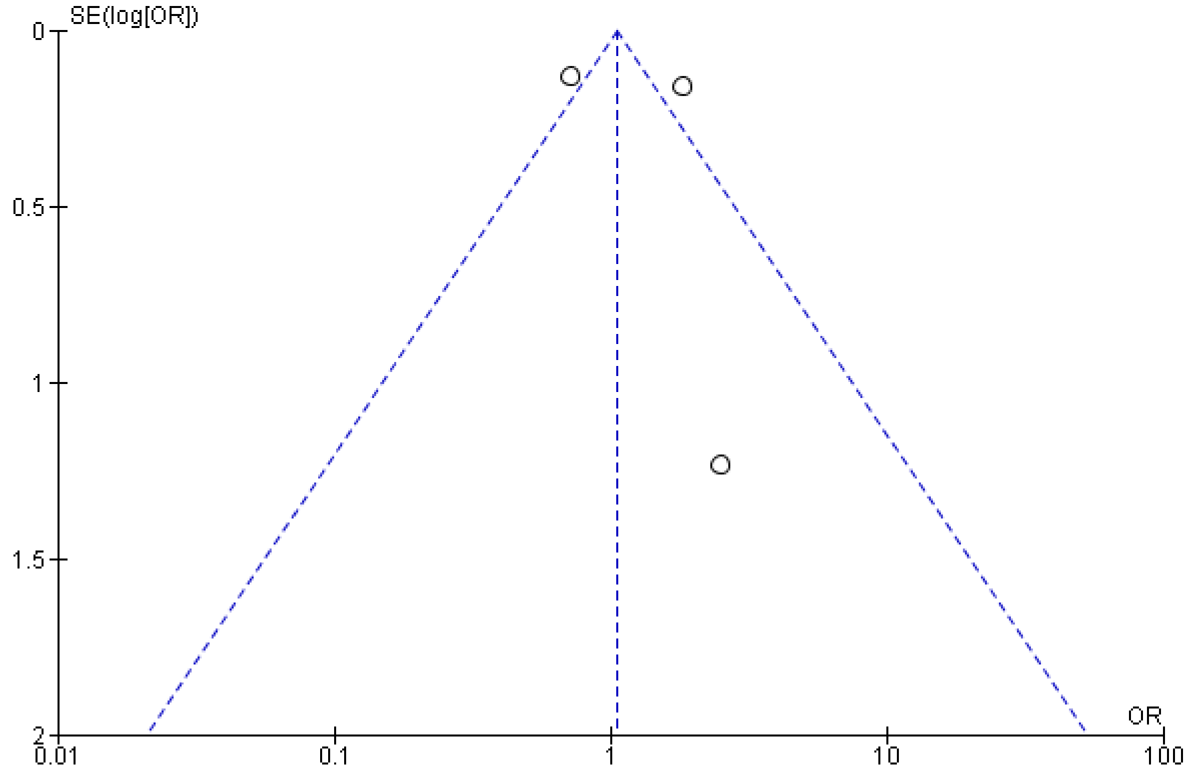

Funnel plot of studies of the GAS5 rs145204276 polymorphism and CRC risk under the recessive genetic model.
