# Supplementary material for: The Role of lncRNA Polymorphisms in Digestive System Cancers: A Systematic Review and Meta-Analysis
Source: Cancers (Basel). 2026 Jun 12;18(12):1916. doi: 10.3390/cancers18121916 (PMC13297490; doi:10.3390/cancers18121916)

Supplementary Figure S3. Forest plots of the association between GAS5 rs145204276 polymorphism and CRC risk under different genetic models: (a) allelic model; (b) dominant model; and (c) recessive model.

a

Study or Subgroup	Case Events	Case Total	Control Events	Control Total	Weight	Odds Ratio M-H, Fixed, 95% CI
Mirea et al., 2025	43	312	29	390	2.0%	1.99 [1.21, 3.27]
Zheng et al., 2016	774	2800	912	2800	58.0%	0.79 [0.71, 0.89]
Zhu et al., 2016	198	1626	555	1852	40.1%	0.32 [0.27, 0.39]
**Total (95% CI)**		**4738**		**5042**	**100.0%**	**0.63 [0.57, 0.69]**
Total events	1015		1496			

Heterogeneity: Chi² = 88.91, df = 2 (P < 0.00001); I² = 98%
Test for overall effect: Z = 9.84 (P < 0.00001)

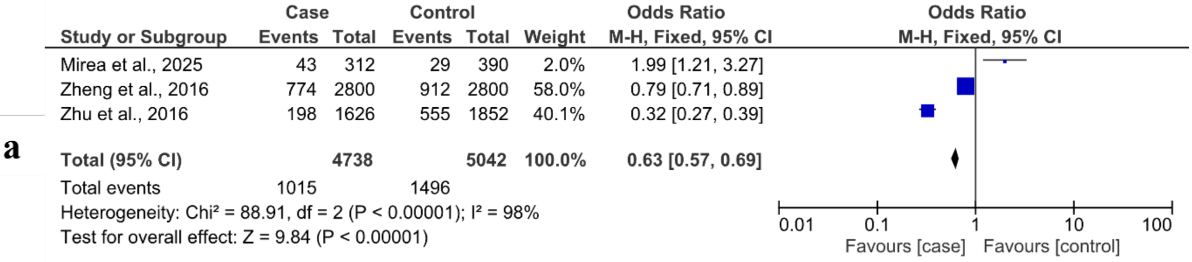

b

Study or Subgroup	Case Events	Case Total	Control Events	Control Total	Weight	Odds Ratio M-H, Fixed, 95% CI
Mirea et al., 2025	41	156	28	195	3.1%	2.13 [1.24, 3.63]
Zheng et al., 2016	662	1400	761	1400	67.4%	0.75 [0.65, 0.87]
Zhu et al., 2016	496	813	482	926	29.5%	1.44 [1.19, 1.74]
**Total (95% CI)**		**2369**		**2521**	**100.0%**	**1.00 [0.89, 1.12]**
Total events	1199		1271			

Heterogeneity: Chi² = 35.66, df = 2 (P < 0.00001); I² = 94%
Test for overall effect: Z = 0.02 (P = 0.98)

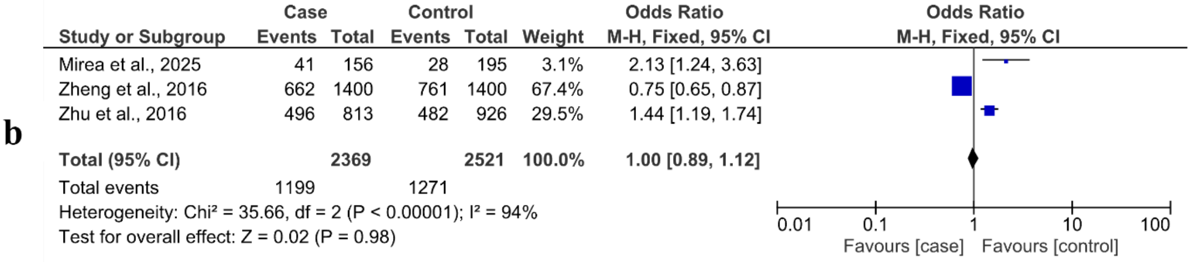

c

Study or Subgroup	Case Events	Case Total	Control Events	Control Total	Weight	Odds Ratio M-H, Fixed, 95% CI
Mirea et al., 2025	2	156	1	195	0.4%	2.52 [0.23, 28.05]
Zheng et al., 2016	112	1400	151	1400	69.8%	0.72 [0.56, 0.93]
Zhu et al., 2016	109	813	73	926	29.7%	1.81 [1.32, 2.47]
**Total (95% CI)**		**2369**		**2521**	**100.0%**	**1.05 [0.87, 1.28]**
Total events	223		225			

Heterogeneity: Chi² = 20.49, df = 2 (P < 0.0001); I² = 90%
Test for overall effect: Z = 0.50 (P = 0.61)

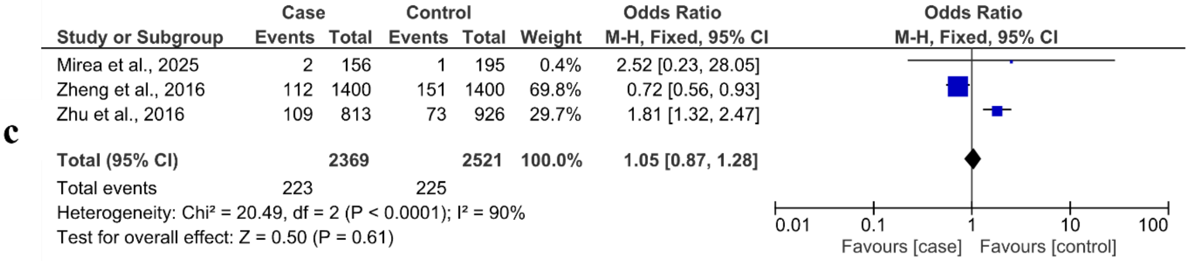

Supplement: Supplementary file 1 [file cancers-18-01916-s001.zip › Supplementary Figure S3.pdf]
